# Essential Oils Applied to Textile Substrates with Emphasis on Antibacterial Properties: Review Article

**DOI:** 10.3390/molecules31071077

**Published:** 2026-03-25

**Authors:** Hendrick Lezeck, Meritxell Martí, Siddanth Saxena, Manuel J. Lis

**Affiliations:** Institute of Textile Research and Industrial Cooperation of Terrassa (INTEXTER), Universitat Politècnica de Catalunya-BarcelonaTECH (UPC), 08034 Barcelona, Spainmeritxell.marti.gelabert@upc.edu (M.M.);

**Keywords:** essential oil, textile substrate, applied methods

## Abstract

Essential oils (EOs) are well-known in traditional medicine, pharmacy, the food industry, and cosmetics because they are readily available and have proven efficacy across a wide range of applications. They are natural, bio-based, and biodegradable, and when applied accurately, they exhibit effective action against microorganisms, viruses, and fungi. However, most organic EOs are volatile and have hydrophobic surface chemistry, making them unsuitable for direct bio-applications in textiles. Textiles offer a useful platform for applying essential oils to impart functions such as antimicrobial or deodorizing effects. While traditional textiles focused mainly on comfort and protection, the rise of functional textiles has created new opportunities to integrate natural compounds such as essential oils. Recently, a growing body of research has focused on integrating essential oils into textile materials, driven by the increasing demand for sustainable fabrics with added biofunctionality. This review highlights the latest advances in applying essential oils to textile substrates and examines the techniques used and the improvements achieved, including washing cycles, antibacterial efficiency ranges, and durability. We survey recent literature, including research papers, articles, and books, to identify the most common methods and clarify their underlying mechanisms.

## 1. Introduction

Essential oils (EOs) have a broad field of application due to their bioactive properties. These applications depend on their quality, source, extraction method, and other factors. They are readily available and have applications across various industries, including food, pharmaceutical, and cosmetic.

EOs have long been used to enhance the taste, aroma, and flavor of food in the food industry [1]. The number of these applications has increased in recent years [2]. Nowadays, researchers are seeking ways to extend the shelf life of foods in packaging using EOs with specific features, because, according to reports from the Food and Agriculture Organization of the United Nations, food spoilage and deterioration of food quality result in the loss of the approximately of 10–40% of all food produced worldwide, with the highest losses occurring in fruit and vegetable crops [3]. This results in a significant loss of both food and economic value. At the same time, an estimated 8.2% of the global population (over 673 million people) experienced hunger in 2024, and 28% were moderately food-insecure, struggling to regularly access adequate food [4]. Researchers realized that EOs contain active compounds (antioxidants) that can fight against microorganisms that cause food spoilage [5]. They know that applying active compounds to food surfaces can pose a risk to people’s health, as some may be allergic to these substances; applying them on packaging becomes an alternative [6].

In the cosmetic industry, EOs are required to provide aroma, meaning they are used in the production of perfumes, soaps, and creams. They have recognized that customer behavior has changed in recent years, with customers seeking high-quality products and products that address environmental concerns. Therefore, new research has been conducted to capitalize on EOs’ use. On the other hand, customers are seeking companies with a sustainable profile [7].

In the case of the medicine and pharmaceutical industry, aromatherapy has been the most crucial application of EOs as medicinal agents for lung disease patients [8]; however, nowadays, medicine has advanced and realized EOs’ potential and has been motivated to figure out ways of working with the active compounds of EOs to fight against microorganisms [9]. The use of traditional medicine has led to increased resistance among microorganisms. Antibiotics that were previously effective against bacteria, fungi, etc., are no longer effective [10]. For example, clove and cinnamon essential oils contain active compounds such as Aldehydes (in Cinnamon) and Eugenol (in Clove), which have proven antimicrobial effects. Aldehydes have a broad-spectrum disinfectant effect that can sterilize and kill fungi, viruses, and bacteria. Eugenol, on the other hand, is a compound shown to have antibacterial properties and may help reduce pain and fight infections [11]. Some EOs come from eucalyptus (*E. glulus*), peppermint (*Mentha pipperita*), anise (*P. anisum*), and sage (*Savia officinallis*), and they are used in medicine as an expectorant for treating cough and bronchitis.

This review aims to cover a wide, dynamically growing field related to the application of EOs on textile substrates to achieve bio-functionality [12], durability, safety, and antibacterial efficiency.

The chemical composition of some EOs, such as citronella, clove, cinnamon, lavender, eucalyptol, thyme, and rosemary, is covered in this review. These oils have been mostly used as antibacterial agents [12]. Terpenes are the primary chemical compounds found in EOs, and their derivatives, terpenoids, possess specific features that can inhibit the growth of microorganisms. Phenylpropanoids are also a crucial ingredient in EOs, and they have antioxidant, antimicrobial, and photoprotective properties, which are highly useful in food preservation, pharmaceuticals, cosmetics, and textile finishing [12,13,14]. Understanding the major chemical constituents of essential oils (EOs) is primordial for interpreting their biological activity.

EOs’ application to textile surfaces is complex but not impossible to achieve practically [15,16,17]. There are examples of companies applying EOs as agents to deliver fragrances, protect our bodies as insect repellents, or combat microorganisms in the environment. Textiles, as the most extensive system that comes into contact with human skin, can serve as carriers for transdermal or dermal delivery of certain active compounds. For example, elderly patients can benefit from clothing, bedding, or other textiles impregnated with aloe vera or similar protective agents during extended periods of bed rest. During laundering, antimicrobial microcapsules or softening agents can be incorporated into fabrics to provide additional comfort and protection. Textiles designed to offer such added biological functions—particularly when combined with natural compounds—are referred to as biofunctional textiles.

“Bio-functional textiles” are products that, in contrast to conventional functional textiles—which provide properties like mechanical strength, moisture management, or thermal regulation—have been intentionally modified to acquire specific biological or bioactive properties, which means that when they are close to the human body they can interact most by delivering active compounds with specific functionality, such as mosquito repellency, drug delivery, cosmetic applications, and sun protection, among others [15,18].

Fabrics are present in numerous situations and may harbor microorganisms that can harm our health. Societal infrastructure, such as hospitals, public transportation facilities, and other key sites, could be prime targets for applying EOs to combat microorganisms [19]. Textile companies, including those involved in spinning, weaving, and finishing, have been encouraged to invest in new technologies to enhance the functionalities of textiles.

## 2. Essential Oils: The Potential Candidates

Essential oils are aromatic and highly volatile liquids obtained from plant material, like bark, seeds, flowers, roots, leaves, fruits, woods, etc. They are well-known in traditional medicine because they are readily available and have demonstrated their efficacy in various fields. Most essential oils are cheaper and easier to procure. Research related to EO use has increased significantly in recent years [7]. However, working with EOs in certain fields, such as the textile industry, is not straightforward, as they are highly volatile and evaporate quickly when exposed to sunlight [12].

Today, EOs are found in a vast number of fields where they are well-suited. Essential oils, when applied accurately, do not damage health; on the contrary, they contain many active compounds that can help combat certain diseases [20,21,22,23,24].

**Figure 1:**
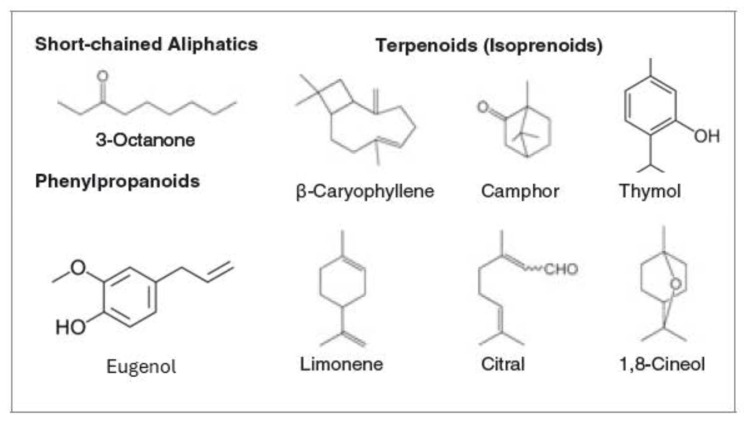
Representative structure for the most useful essential oil organic compounds [26].

The vast majority of EO are made by lipophilic terpenoids, phenylpropanoids, or short-chain aliphatic hydrocarbon derivatives [25] of low molecular weight. The most representative structures are shown in Figure 1.

### 2.1. Chemistry of Essential Oils with Antibacterial Properties

Due to the several chemical compounds inside essential oils, the Food and Pharmacy Industry, the Cosmetic field, and the Textile industry are very keen to find a way to work more deeply with EOs, because they are very well-known for traditional medicine, and they cover a considerable number of applications in these fields. Therefore, this review will focus on working with citronella, cinnamon, clove, eucalyptus, thyme, and rosemary.

The selected essential oils (Table 1) are chemically characterized by a predominance of oxygenated monoterpenes (e.g., 1,8-cineole, linalool, geraniol, citronellal) and phenylpropanoids (e.g., cinnamaldehyde and eugenol). Phenylpropanoid derivatives, particularly cinnamaldehyde and eugenol, exhibit strong bactericidal effects due to their reactive aldehyde and phenolic groups, which promote membrane disruption and protein denaturation. In contrast, oxygenated monoterpenes mainly act by increasing membrane permeability and disturbing cellular homeostasis. The overlapping presence of lipophilic terpenoid structures across these oils explains their broad-spectrum antimicrobial activity and their suitability for incorporation into functionalized textile systems designed for controlled bioactive release [27,28,29]. Moreover, these EOs have been studied in the context of functionalized fabrics and encapsulation systems, where they retain antimicrobial activity relevant to biofunctional textiles [30]

### 2.2. Antibacterial Activity of Essential Oils

Among EOs reported in the literature, clove, thyme, rosemary, citronella, and lavender are often known to exhibit low MIC (Minimum Inhibitory Concentration) against both Gram-positive and Gram-negative bacteria (Table 2), due to their high content of phenolic and aldehydic compounds (e.g., thymol, eugenol, cinnamaldehyde). In contrast, oil compositions by hydrocarbon terpenes generally show weaker antibacterial performance. Therefore, these selected EOs represent rational candidates for antimicrobial action [28,31].

### 2.3. Essential Oils with Properties for Combating Micro-Organisms

#### 2.3.1. Citronella Essential Oil

Citronella oil (*Cymbopogon nardus*) is a well-known essential oil readily obtained by steam distillation of the plant. It is an aromatic plant, and its oil has health-beneficial properties. Citronella oil is used for treating rashes and certain infections, and it also serves as an insect repellent. The dual functionality of insect repellency and antibacterial activity in textiles (Table 3) inherently involve performance trade-offs. Repellent efficacy relies largely on the volatility and diffusion rate, ensuring enough airborne concentration to disrupt insect host-seeking behavior [33]. On the other hand, antibacterial action depends on a direct contact mechanism, to be able to provoke the membrane disruption, the leakage of intracellular components, and metabolic inhibition. Those mechanisms require the retention of active molecules at the textile–bacteria interface [34]. Therefore, increasing volatility to enhance repellent performance may accelerate the depletion of active compounds, reducing long-term antibacterial activity. Conversely, enhancing encapsulation density or reducing release rate to prolong antibacterial activity may limit vapor-phase concentration, weakening repellency.

Although this essential oil has a beneficial effect against insects, its application is limited in the washing durability across multiple cycles. There are examples of good results by applying citronella EO to textile substrates using microcapsules as carriers. Zeeshan Tariq [35] reported promising results when preparing microcapsules of citronella oil via complex coacervation and applying them to polyester/cotton (PC) with glutaraldehyde as a stabilizer and an acrylic-based binder to enhance washing durability. The fabric sample finished with 15% (solid in water) citronella oil microcapsules. At zero washes, the sample exhibited 90% mosquito-repellency, and after 30 washes, the fabric still possessed 80% mosquito-repellency, confirming the durability of the as-prepared finished fabric.

Lis Arias [18] developed citronella oil microcapsules as a natural insect repellent using the complex coacervation method with Gelatin and Arabic gum and applied them to cotton/polyamide fabrics to control drug delivery. The retention capability of the microparticles was assessed using an in vitro experiment. The results showed long-term dosing capability and a promising application for home use and clothes, in environmental conditions, for insect control.

Bancha Yingngam [36] employed a two-step approach to produce citronella microcapsules, which involved oil-in-water emulsification and spray drying, to encapsulate citronella oil in acacia gum microcapsules for possible application in a nonwoven (not informed composition) cosmetic textile. According to the results assessed by naked eyes, microcapsules obtained by spray drying improved the thermal stability, decreasing the irritation potential of citronella oil compared with the thermal stability and irritation potential of pure EO. The resulting microcapsules have the potential to offer prolonged odor volatility with reduced irritation, which allows for further development of citronella essential oil-based cosmetic textiles.

#### 2.3.2. Clove Essential Oil

Clove is an angiospermic plant and belongs to the division of *Magnoliophyta* in the kingdom *Plantae*. The tree grows primarily in Indonesia, India, Madagascar, Zanzibar, Pakistan, and Sri Lanka. The chemical composition of clove essential oil is complex. It can vary depending on the location and the method used to extract them. Omidbeygi et al. [37] reported the general composition of clove essential oil (cultivated in Iran and extracted for 3 h by distilled water) to be mainly eugenol (63.37%), β-caryophyllene (15.94%), eugenyl acetate (13.14%), α-humelene (2.62%), and caryophyllene oxide (1.06%).

Due to eugenol being the main active compound in clove oil (Table 3), this essential oil possesses pharmacological properties such as antimicrobial, anti-inflammatory, analgesic, neuroprotective, and antitumor activities, making it a versatile natural ingredient that can aid in the prevention and treatment of several disorders. Varma [38] prepared clove EO by complex coacervation using five different EO concentrations and applied it to cotton fabric to analyze antibacterial activity using standard parameters. The zone of inhibition was found to be maximum at high clove concentration on treated cotton fabric before wash with 12.5 mm ± 1.5 and 12.33 ± 0.57 against *S. aureus* and *E. coli*. Cotton treated with MCCL (microcapsule clove oil) showed good wash durability against *S. aureus* as compared to *E. coli.* The antimicrobial property decreases with increasing number of wash cycles.

Sharma [39] manufactured clove EO microcapsules by spray-drying in a chitosan biopolymer to enhance antibacterial activity on cotton fabrics via the padding technique using three different cross-linkers, viz., malic acid, maleic acid, and Eudragit S100. Eudragit S100 gave the best results among the other cross-linkers, with a 98.5% reduction in bacterial CFU, compared to 95.3% with malic acid and 91.8% with maleic acid.

#### 2.3.3. Cinnamon Essential Oil

Cinnamon (*Cinnamomum zeylanicum*) is among the earliest spices used in food. Cinnamon is readily available in its natural form and is also commercially produced, typically as dry or milled bark. Bark oil, bark oleoresin, and leaf oil are essential value-added products of cinnamon. Bark oil is used in the food and pharmaceutical industries.

According to Cardoso-Ugarte [40], several methods are available for extracting oil from plants, including solvent extraction, microwave-assisted extraction, and hydrodistillation.

Cheng et al. [41] provide detailed information related to the characterization of the compounds of the cinnamon essential oil, typically carried out using gas chromatography–mass spectrometry (GC–MS) to identify diverse volatile components. The main compounds found in cinnamon essential oil are diterpenes, hydrocarbons, oxygenated diterpenes, monoterpenes, and oxygenated monoterpenes.

Cinnamon was used in the food industry as a spice. Nowadays, it has been granted “generally recognized as safe” status as a food additive by the Food and Drug Administration. It is useful as a food preservative to inhibit fungal growth [42] and, owing to its antioxidant properties and antibacterial, insecticidal, and nematocidal activities, has been widely used in the food industry. Additionally, it is used in seasonings, sauces, bakery products, confectionery, and beverages.

On the other hand, many studies have developed new applications for cinnamon essential oil in the pharmaceutical industry (Table 3). Research has shown that essential oils and their components also have antimicrobial, insecticidal, acaricidal, antityrosinase, antioxidant, and antimutagenic properties [43].

Cinnamon, along with other essential oils, should be consumed in very small doses because certain cinnamon compounds can be detrimental to health. Their antibacterial and repellent efficacy often requires relatively high concentrations, raising concerns about toxicity and safety [44]. Several EO constituents, particularly phenolic compounds such as eugenol, thymol, and cinnamaldehyde, exhibit dose-dependent cytotoxicity, skin irritation potential, and sensitization effects when applied at elevated concentrations. For example, cinnamic acid, a compound used in perfumery, can cause dermatitis and, when added to toothpaste, can promote oral sensitivity. In textile applications, prolonged dermal exposure may increase the risk of irritation, especially under occlusive conditions [43]. Moreover, high volatile organic compounds (VOC) may raise inhalation safety considerations [45]. Strategies such as microencapsulation, controlled-release systems, and reduction in free oil concentration through synergistic combinations can mitigate the risks by lowering the direct exposure while preserving functional performance [43].

Nagender Singh and Javed Sheikh [46] encapsulated cinnamon bark oil in a gelatin-chitosan complex using optimized spray-drying technology, resulting in spherical, solid, and micro-sized capsules. The goal was to apply the linen fabric using the pad-dry method and to analyze the finished fabric’s functional properties, including antioxidant, antibacterial, fragrance supplier, and mosquito-repellent activities. The effect of laundering on the functional properties of finished linen fabric was also studied. The microcapsules possessed high efficiency (78.67%) and irregular size and shape. The microcapsules adhered to the textile surface via ionic interactions, facilitated by a chitosan–acrylic binder. The stable microcapsules enabled slow release of the active compound on the textile surface. Their antibacterial activity was attributed to diterpene groups, which can protect against various biological agents [46,47]. The treated fabric exhibited antibacterial activity against *E. coli* and *S. aureus*, excellent mosquito repellency (up to 100%), durability through ≈ approximately 20 washes, and a pleasant fragrance finish.

#### 2.3.4. Lavender Essential Oil

The essential oil from the *Lavandlula genus* has been used for centuries in traditional medicine as a therapeutic and aromatic agent due to its carminative, sedative, and antidepressant properties, and has gained popularity in the flavor and fragrance industries [48].

Nowadays, the pharmaceutical industry has recognized that certain bacteria and viruses have developed strong resistance to traditional antibiotics. Due to its antimicrobial, antioxidant, antifungal, insecticidal, and insect-repellent properties, it has emerged as a promising candidate for supplementing or replacing certain synthetic treatments [48].

The food industry has increased the use of lavender essential oils because they are effective at controlling bacterial growth in food products. According to reports from the Food and Agriculture Organization of the United Nations, food spoilage and deterioration of food quality result in the loss of 10–40% of all food produced worldwide, with the highest losses occurring in fruit and vegetable crops [3]. However, synthetic additives used to control spoilage may pose health and environmental risks. Lavender EO has shown efficacy against *Escherichia coli*, a major contaminant in the meat-processing industry.

Evidence from “in vitro” studies suggests that lavender EO can be effective against a wide range of food-borne pathogens, including *Salmonella*, *E. coli*, and *Enterobacteriaceae*, at levels as low as 10% [49]. In cases where direct application to food is impractical—for example, fish—a biodegradable gelatin–chitosan film containing lavender EO has been developed and applied successfully [50].

Lavender essential oils have shown excellent insect-repellent properties. Perhaps, among its applications in various fields, the insect repellency is the most notable. Lavender EO and its constituents alone have been tested in insect repellency and toxicity assays and have been effective [51]. Generally, monoterpene ketones exhibit higher activity than alcohols, with some of the most potent monoterpenes including terpineol, camphor, and cineole [12].

In the textile field, lavender essential oils have the most significant application as fragrances. The aim of Shuo Wang’s work [52] was to increase the time of fragrance action on the textile surface. But, as is well known, lavender is similar to other essential oils and is very volatile and sensitive to sunlight. Therefore, microcapsules were chosen as the most appropriate carriers for lavender. Lavender EO microcapsules were produced via interfacial polymerization using polyurea as the shell material. The polyurea shell was formed using hexamethylene diisocyanate and guanidine carbonate, thereby eliminating the need for a crosslinking agent. The study reported good fragrance-retention results over time. After twelve weeks, fabrics treated with lavender EO microcapsules showed better sustained-release properties than those treated with a fragrance emulsion, as measured by gas chromatography.

Beyond its aromatic properties, the antibacterial performance of lavender EO is well documented [50] and attributed mainly to monoterpenes, alcohols such as linalool and linalyl acetate (Table 2), which can disrupt bacterial membrane integrity. Balasubramanian [53] used electrospinning to encapsulate lavender EO in polyacrylonitrile (PAN) nanofibers. Antibacterial proficiency was assessed by challenging the material against *E. aureus* and *K. pneumoniae*. The incorporation of lavender EO exhibited effective antibacterial properties, with a zone of inhibition of 14–15 mm for at least 8 h, and it remained unaltered over 30 days. Upadhyay [54] encapsulated lavender EO via complex coacervation and applied it to woven linen to analyze the antibacterial effect against *E. aureus* and *K. pneumoniae*. The fabric samples 1 analyzed showed significant antibacterial inhibition (25 and 20 mm, respectively), and sample 2 showed exceptional antibacterial activity against *Enterococcus*, with inhibition zones of 30 and 27.8 mm.

#### 2.3.5. Eucalyptus Essential Oil

Eucalyptus essential oil is derived from several plant families, including *Poaceae*, *Lamiaceae* (Labiatae), *Zingiberaceae*, *Rutaceae*, *Asteraceae* (Compositae), and *Myrtaceae*. The genus Eucalyptus comprises more than 500 species, many of which originate from Australia and Tasmania. One of the most widely studied species is *Eucalyptus citriodora*, known for its high natural variability and the high quality of its essential oil, which contains citronellal as the major component. Eucalyptol EO has important applications in the pharmaceutical, food, and cosmetic sectors (Table 3). Ramezani [55] reported that eucalyptus significantly reduced the mycelial growth of rice pathogens.

A recent report by Manzoor [56] identified terpinen-4-ol in eucalyptus EO. This compound exhibits insecticidal activity and has been reported to induce apoptosis in lung cancer cells both in vitro and in vivo. Furthermore, eucalyptus EO has shown activity against a range of Gram-positive and Gram-negative microorganisms, including *Salmonella typhi*, *Bacillus subtilis*, *Staphylococcus aureus*, *Pseudomonas aeruginosa*, *Escherichia coli*, and *Klebsiella pneumoniae*, as well as *Candida albicans* [57,58,59].

The most significant textile application of eucalyptus EO has been achieved through microencapsulation. Joo Ran Kim [60] developed eucalyptus EO-loaded microcapsules via coacervation to create environmentally friendly and acaricidal fabrics for controlling house dust mites. The microcapsules were grafted onto cotton fabrics, providing acaricidal activity. Glutaraldehyde was used as a crosslinking agent to stabilize the shell material during the gel stage. Mortality of house dust mites (HDMs) in contact with the treated fabric was evaluated according to AATCC Test Method 194-2007. Results showed that fabrics grafted with eucalyptus oil microcapsules (EOMCs) exhibited strong biological activity, achieving 98.7% mortality of HDM. Moreover, Benas [61] successfully incorporated eucalyptus EO microcapsules, prepared via the layer-by-layer methodology, into nanofibers via electrospinning to manufacture medical masks (Personal Protective Equipment). The goal was to produce odorless masks using aromatic EO. The work obtained good results, proving that this type of mask’s middle layer effectively protects against coronavirus and provides better scents.

#### 2.3.6. Thyme Essential Oil

Thyme (*Thymus vulgaris* L.), belonging to the *Lamiaceae* family, is a perennial subshrub with a lifespan of approximately 10–15 years. Its stem becomes woody with age, and it exhibits both horizontal and upright growth habits [62]. Thyme is cultivated worldwide. The plant can be propagated by seeds or by vegetative parts, such as root cuttings; the latter method has been well described by Ozguven and Tansi [62].

Thymol and carvacrol are major constituents of thyme EO. These compounds have important applications in the pharmaceutical and food industries due to their antimicrobial and antioxidant properties (Table 3). The components of thyme EO may act individually or synergistically, contributing to its broad biological activity.

Thyme EO was initially used to enhance the flavor and aroma of food. The antioxidant constituents in thyme EO are important for extending shelf life by protecting food from oxidative processes. Szczepaniak et al. [63] reported that a mixture of thymol, carvacrol, and cymene inhibited the growth of *Brochothrix thermosphacta*—a common spoilage organism in meat stored under high-oxygen modified atmospheres—by 25.7%, thereby extending the shelf life of minced pork during the study.

The pharmaceutical industry has also explored the antimicrobial and antioxidant properties of thyme EO. Kon and Rai [64] created blends of thyme, cinnamon, and rose EOs. A mixture of thyme and cinnamon EO showed synergistic antibacterial activity against *S. aureus*, while a blend of thyme and rose EO showed strong activity against *E. coli* [64].

In the textile industry, microencapsulation is the most common method for applying thyme EO to fabrics. Liliana Indrie [65] investigated thyme EO treatments to improve the mechanical properties of heritage textiles (cotton and hemp) after long-term museum exposure. Over time, these textiles showed reduced mechanical strength. Thymol from thyme EO was encapsulated in ethyl cellulose (49% ethoxy content) via the solvent evaporation method. Ethyl cellulose was selected as the shell material because it is commonly used to control the release of active compounds in pharmaceutical tablets [49,66]. The treatment resulted in significant improvements in tensile strength, suggesting applicability in museums, universities, and ethnographic collections.

Rose [67] applied thyme EO to wool fabrics for aromatherapy purposes. Microcapsules were prepared via complex coacervation and applied to wool textiles to deliver therapeutic effects and a long-lasting aroma. The durable fragrance retention was attributed to wool’s high moisture absorption and the incorporation of β-cyclodextrin as a binder to enhance adhesion between the microcapsules and the fabric.

Subair [68] encapsulated thymol and carvacrol with chitosan and applied the resulting microcapsules in the cotton textile to manufacture laboratory coats (Personal Protective Equipment). The goal was to prevent direct exposure to pathogens, bacteria, spills, and burns. The treated fabrics achieved up to a 4-log reduction (99.99%) in human pathogens and retained antibacterial activity even after multiple wash cycles. While strong antimicrobial effects have been reported for eucalyptus and thyme EOs, it is crucial to recognize that interactions with the binder and encapsulating matrix may influence their performance in textiles. Textile binders such as chitosan, ethyl cellulose, β-cyclodextrin, or crosslinked polymers can modify the release kinetics of volatile compounds, thereby affecting surface availability and biological efficacy. In some cases, synergistic effects may arise when the binder itself exhibits antimicrobial activity, as observed with chitosan-based systems [69]. On the other hand, highly crosslinked or hydrophobic matrices may delay diffusion of active compounds such as thymol or terpinen-4-ol, potentially decreasing immediate antimicrobial effects [34]. Therefore, the overall bioactivity of EO-functionalized textiles depends not only on the intrinsic potency of the oil but also on matrix–compound interactions and controlled-release behavior.

#### 2.3.7. Rosemary Essential Oil

The use of EOs has increased, particularly for oil from the rosemary plant (*Rosmarinus officinalis* L.), because this EO exhibits antimicrobial, antifungal, and antioxidant properties, and, above all, is low-cost and readily available [70]. The genus *Rosmarinus* (family *Labiatae* or *Lamiaceae*) comprises three different species (*Rosmarinus officinalis*, *Rosmarinus eryocalix*, and *Rosmarinus tomentosus*) found primarily in the western Mediterranean region, and Central and South America. Its natural habitat spans from areas close to the sea up to 1500 m above sea level [70]. The chemical composition of rosemary oil has been widely studied. According to Napoli [71], rosemary EO can be classified as one of three chemotypes: cineoliferum (high 1,8-cineol content), camphoriferum (camphor > 20%), or verbenoniferum (verbenone > 15%). Today, rosemary EO is a product with good prospects for the pharmaceutical, cosmetic, and food industries, endowed by its chemical composition with beneficial properties that meet society’s growing demand for natural products [72]. The latest research on the use of rosemary EO for medical purposes has mainly focused on its antibacterial, antifungal, insecticidal, anti-inflammatory, and other properties (Table 3). As an antibacterial agent, rosemary EO has been tested against pathogenic microorganisms such as *E. coli*, *Bacillus cereus*, and *Staphylococcus aureus* [73]. Gram-positive bacteria are more sensitive to EO, as the hydrophilic cell wall structures of Gram-negative bacteria have been demonstrated to block the penetration of hydrophobic components through the cell membrane. Finally, in the textile field, which is the area focused on in this paper review, Singh [74] applied rosemary to linen fabric, resulting in durable antibacterial activity, significant antioxidant activity, mosquito repellency, and pleasant aroma.

In summary, the performance of essential oils improves when they are protected from environmental factors.

**Table 3:** Summary of typical essential oils with their respective functional ingredients for possible applications.

Type	Botanical Name	Functional Constituent(s)	Properties	Applications	Reference
Agarwood	*Aquilaria malaccensis*	Plyphenols	Antioxidant and antimicrobial	Food industry and pharmaceutical industry	Piah [75], M Zhou [76]
Black pepper	*Piper nigrum* L.	Piperine	Antioxidant and antimicrobial	Food preservatives, pharmaceutical industry	de Almeida [66], Bastos [77]
Cinnamon	*Cinnamon zeylanicun*	Cinnamaldehyde, camphor, eugenol	Antioxidant and antimicrobial	Textile and food industry	de Almeida [66], Ghayempour [16], Jiang [78], de Souza [79], Singh [80]
Citronela	*Cymbopogon nardus*	Citronellal, Geraniol, Citronellol	Antioxidant and antimicrobial	Personal care, household products, pharmaceutical, and Food Industry	Tariq [35], Specos [81], Lis Arias [82], Yingngam [36], Liu [83]
Clove	*Syzygium aromaticum*	Eugenol, carvacrol, thymol	Antioxidant, antimicrobial, and aroma	Cosmetic, food, and pharmaceutical industry	de Almeida [66], Ghayempour [16], El Molla [84]
Eucalyptus	*Eucalyptus citriodora* Hook	Citronellal	Antioxidant and aroma	Cosmetic and pharmaceutical industry	Kim [60], Elbhnsawi [85]
Garlic	*Allium sativin* L.	Sulfur	Antimicrobial and antioxidant	Food, medicine	C W Tsai [86], Chung [87], Park [88]
Lavender	*Lavandula angustifolia*	Camphor, Linalool, 1,8-cineole	Antifungal, antimicrobial, and antioxidant	Sedative, antidepressant, carminative	Ghayempour [16], El Molla [84], Golja [89], Wang [52]
Lemon Grass	*Cymbopogon* spp.	Citral	Frangrance, Aroma, Mosquito Repellent	Food industry, retard microbial activity	Miro Specos [90], Bhatt [91]
Lime	*Citrus limon*	Limone, Citral, Terpenes	Flavor (bioactive) antiseptic, antioxidant	Food, medicine, sedatives, and aromatic	Julaeha [92]
Neem	*Azadirachta indica*	Flavonoids, phenolics, steroids	Antimicrobial, anti-oxidant, immunostimulant	Acaricidol, Mosquito Repellent	Sayed [93]
Oregano	*Origanum* spp.	Thymol, carvacrol, p-cymene	Anti-microbial and anti-oxidant	Flavor, season agents (food industry)	Wu [94], de Almeida [66]
Rose	*Rosa x damascene Mill*	citronella, geraminol, nonadecane	Anti-oxidant and antimicrobial	Flavoring agents, pharmaceutical	Golja [89], Stan [17]
Thyme	*Thymus vulgaris* L.	Thymol and carvacrol	Anti-microbial and anti-oxidant	Food (beverages) and pharmaceutical	de Almeida [66], Ghayempour [16], El Molla [84]
African cardamon	*Aframomum danielli*	1.8 cyneole, β-pinene, α-terpineol	Anti-oxidant and antibrowning	Food preservative (controlling microbial deterioration)	Martins [95], Adegoke [96]
Amazon Rosewood	*Aniba rosaedora*	Linalool, β-phelladrene	Anti-oxidant	Flavor and cosmetic industry	López [97], Belletti [98]
Angelica	*Angelica glauca*	Methyl-octane, limonene, trans-carcavol	Anti-oxidant, anti-microbial activity	Food and preservatives	Kandari [99]
Jasmine	*Jasminun sambac*	Linalool, (monoterpenoide alcohol), benzula	Anti-microbial and anti-oxidant	Food preservation and flavor agent	F Abdoul-Latif [100], E Hernandez [101]
PepperMint	*Mentha spicata* L.	Tepernoides (derivade from isoprene), carvone and cimonene	Anti-oxidant, anti-fungal and aroma	Pharmaceutical, perfumery, and food industry	L Ye [102]
Onion	*Allium cepa*	Sulfur (Dipropil dissulphyde)	Anti-microbial, sensorial and anti-oxidant	Food preservative, anti-rancidity	Benkeblia, N [103]
Rosemary	*Rosimarinus officinalis*	Canphor, 1.8 Lineol	Anti-microbial and anti-oxidant	Preservative food.	Golja [89]

## 3. Methods to Immobilize the Essential Oil

To protect the chemical compounds inside EOs, they must be immobilized in a suitable location, preferably in conjunction with the substrate [14,104]. The primary reason is the immobilization of the whole chemical compounds from EOs. This protection can offer multiple benefits and various applications. The material immobilized exhibits antibacterial properties, and the prolonged diffusion of the chemical compounds contained inside EOs from the tissue to the skin of the patient can be detected, leading to the reservoir effect.

There are many immobilization processes; however, not all of them are recommended for use on textile surfaces [13,14,105]. Therefore, choosing the method that best fits for immobilizing chemical compounds from EOs can be a challenging task.

The choice of shell material is the initial phase, and selecting the most appropriate immobilization method to protect organic compounds from EO is a two-step process. The shell material will directly influence the stability of the chemical compounds in the core. This choice should take into account two crucial features that will play a vital role in the capsules/complexes formed [14,105]:

The first step is selecting the raw material for the shell. A wide range of synthetic and natural materials is available for wrapping chemical compounds; however, this review aims to identify the most common shells for working with EOs to achieve stability and biodegradability, and to apply these complexes/capsules to textile substrates [14,105].

The second step concerns the shell’s functionalities in contact with the tissue. Several key factors must also be considered, but the most crucial decision pertains to the ultimate application of the fabrics. For instance, to match the criteria of environmentally friendly, or permanent protection, or separation of core material for the life of the product, or targeted release of the core under planned conditions that trigger the opening of the shell, or finally, long-lasting and gradual release by diffusion through the permeable microcapsule shell [14].

### 3.1. Complex Formation by Synthetic Polymers

The most common synthetic polymers used to immobilize essential oils are as follows [105]:

- Polyvinyl alcohol (PVA): often used as a stabilizing agent during the encapsulation process.
- Polylactic acid (PLA): biodegradable and suitable for controlled release applications.
- Polymethyl methacrylate (PMMA): provides good mechanical strength and stability.
- Polyurethanes: used for forming strong, stable capsules.
- Polycaprolactone (PCL): a biodegradable polymer used for long-term controlled release.

These polymers generally have high molecular weight and, being synthetically derived, are not classified as biopolymers [106]. Research in microbial biodegradation aims to address the limited degradability of such polymers. Danso et al. [107] reviewed current knowledge of microbial plastic degradation, showing that certain microorganisms and enzymes can degrade some synthetic polymers, including polyethylene terephthalate and ester-based polyurethanes.

### 3.2. Complex Formation by Using Biopolymers: Chemical Methods

One of the major challenges in the textile industry is the development of biodegradable complexes for fabric applications [108]. This demand has increased as consumers now seek textiles with enhanced performance rather than basic functionalities such as design or skin protection. This trend has encouraged companies to develop functional textiles with improved properties [108].

Biodegradable microcapsules, cyclodextrins, and liposomes are among the techniques that can endow textile substrates with biofunctionality [109,110,111]. However, there is a shortage of technologies capable of producing fully biodegradable complexes. A primary limitation is that many conventional chemical polymerization methods are not compatible with biodegradable biopolymers. As a result, biodegradable complexes may exhibit reduced fabric performance due to these chemical constraints.

Biodegradable polymers may be derived from natural sources or synthesized chemically. The presence of heteroatoms in their main chain makes them susceptible to hydrolytic cleavage of ester (–COO–), amide (–CONH–), or ether (–O–) bonds. Natural biodegradable polysaccharides include cellulose, chitin, chitosan, amylose, sodium alginate, and lignin. Other biodegradable polymers include amide-containing polymers (such as polypeptides, proteins, and thermal polyaspartate), biodegradable polyurethanes, and polyesters such as polycaprolactone (PCL), polylactic acid (PLA), poly(3-hydroxybutyrate), and polyhydroxyalkanoates (PHAs), as well as their copolymers.

Although natural polymers such as chitosan, cellulose, alginate, and Arabic gum are often described as sustainable alternatives to synthetic polymers, their sustainability must be evaluated beyond material origin. A comprehensive sustainability assessment should consider life-cycle factors, including raw material sourcing, energy consumption during processing, solvent use, cross-linking agents, scalability, durability, and end-of-life biodegradability. Therefore, in the context of this review, the term “bio-based” refers to systems derived from renewable resources, while acknowledging that full sustainability evaluation would require life-cycle assessment (LCA) studies [112,113].

#### 3.2.1. Chitosan

Chitosan is among the most widely used biopolymers for encapsulation. It is relatively inexpensive, readily available, and its cationic nature is important for antimicrobial activity. Chitosan can be combined with various other biopolymers and meets many industrial requirements, including scalability.

This biopolymer has received significant attention in complex formation, and the main advantages of chitosan-based microcapsules as drug carriers are their controlled-release properties and biocompatibility [114]. Chitosan is also considered a suitable wall material for encapsulation in textile-finishing applications [59]. The active compound (EO) can be delivered to the textile surface by modifying the shell permeability or through external stimuli such as temperature, pressure, or pH, which induce swelling and diffusion of the core material.

Currently, chitosan is widely used in the textile industry due to its antibacterial activity, coloration properties, UV resistance, and thermal stability. Its hydrophilic nature and cationic charge in acidic media enable the development of mild microencapsulation methods to obtain micro- or nanoparticles.

**Figure 2:**
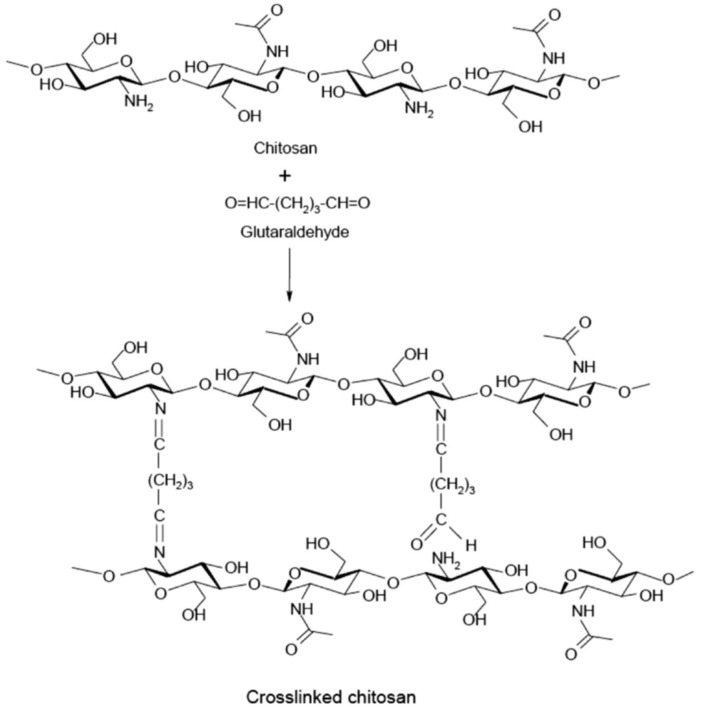
Chitosan molecules reacting with glutaraldehyde to form cross-linked chitosan (reprinted with permission from Yang et al., 2004) [115].

Chitosan is obtained by deacetylating chitin, which is sourced from crustacean shells or fungal materials, and may exhibit different behaviors under different pH conditions [95,96]. Its chemical structure is well established: chitosan is a linear polysaccharide composed of glucosamine and N-acetylglucosamine units (Figure 2). The amino groups (–NH_2_) can be protonated in acidic environments, endowing chitosan with a cationic character that contributes to the antibacterial performance of EO-loaded microcapsules.

Chitosan has many advantages, including its biodegradability and antimicrobial properties. However, several drawbacks may limit its large-scale application, such as limited shelf life, relatively poor mechanical properties, the need for chemical modification, and cost considerations. Researchers are addressing these limitations by developing chitosan derivatives with improved performance or by combining chitosan with other polymers to enhance its functionality.

#### 3.2.2. Gelatin-Based

Gelatin is a natural polymer derived from collagen and can be sourced from protein-rich animal materials such as skin, bones, connective tissues, fish scales, and even insects. This biopolymer has a strong capacity to form films, making it highly suitable for producing microcapsules and for developing stimuli-responsive systems compatible with human skin.

**Figure 3:**
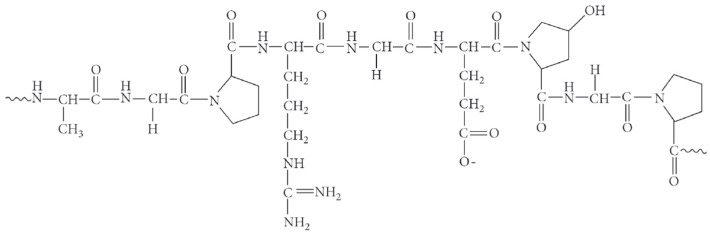
Chemical structure of gelatin [116].

Because gelatin chains are composed of 18 amino acids linked by peptide bonds, the polymer can retain water and easily form stable films (Figure 3). These characteristics make gelatin a promising wall material for microencapsulation.

Gelatin-based systems, due to their biocompatibility, biodegradability, and low immunogenicity, can be combined with several natural and synthetic polymers, including chitosan, alginate, fibrinogen, and sericin. Studies on chitosan–gelatin complexes prepared at different ratios have shown that chitosan can significantly improve the tensile strength of the complexes in both dry and swollen states. Chitosan also reduces water uptake, indicating strong interactions between the rigid chitosan chains and the more flexible gelatin chains, replacing polymer–water interactions [116].

**Figure 4:**
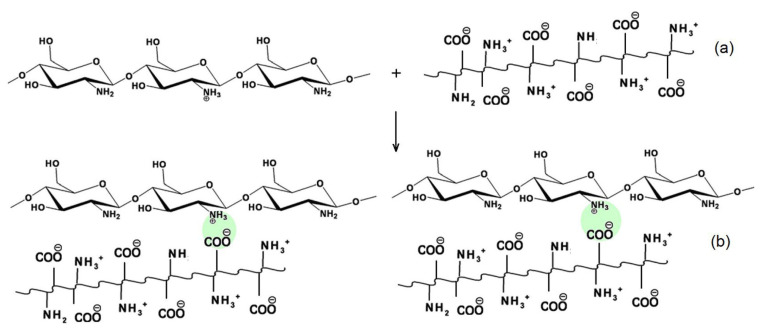
Possible reaction mechanisms: (**a**) chitosan and gelatin microstructure, and (**b**) ion exchange in acetic acid and a possible reaction mechanism between chitosan and gelatin [117].

Samimi Gharaie [117] evaluated the combination of these two polymers and ionic interaction between positively charged chitosan and negatively charged gelatin, using chitosan–gelatin complexes (Figure 4). Figure 4 shows that in the spectrum of chitosan/gelatin, the C=O groups of gelatin are adsorbed with the N-H groups of chitosan, resulting in strong hydrogen bonds, leading to a far more miscible chitosan gelatin component. Their results confirmed previous findings: increasing the chitosan content enhanced the mechanical strength of the complexes and reduced water absorption due to stronger intermolecular interactions between the two polymers.

They also studied the influence of crosslinking on the microcapsules’ performance. The controlled transport of essential oil was successfully achieved, and the thermo-responsive behavior of gelatin-based microcapsules supported their potential as temperature-responsive delivery systems for textile and biomedical applications [118].

**Figure 5:**
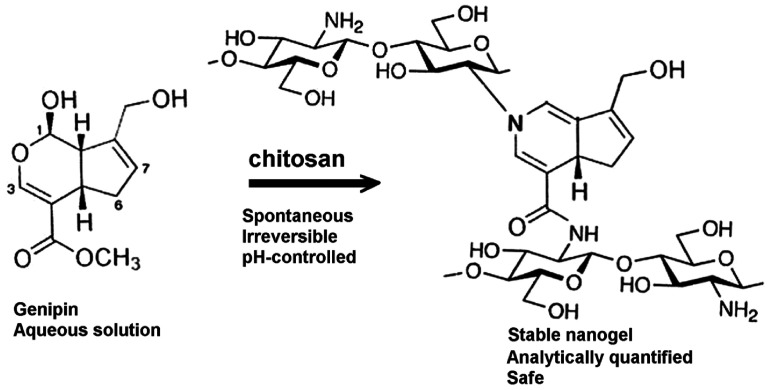
Two chitosan chains cross-linked by one mole of genipin, forming a monosubstituted amide and a tertiary amine [120].

Hussain and Maji [119] studied the microencapsulation of *Zanthoxylum limonella* oil (ZLO) using chitosan–gelatin complexes crosslinked with genipin (Figure 5). They observed that increasing the chitosan content improved both the thermal stability of the microcapsules and the control over oil release.

Gelatin has a high affinity for water, giving it physical properties similar to those of living tissues, such as low interfacial tension and good compatibility with aqueous environments. As a result, gelatin-based microcapsules possess well-suited attributes for biomedical applications.

Another key advantage of gelatin is its ability to exhibit *stimuli*-responsive behavior—particularly thermoresponsiveness—which allows it to work effectively at human skin temperature. These properties also make gelatin useful for fragrance delivery. For example, Rungwasantisuk et al. [121] produced aromatic microcapsules containing lavender essential oil (LEO) by complex coacervation using gelatin and gum Arabic as shell materials. The microcapsules were added to a UV-curable varnish for screen printing on gift-wrapping paper. Burst release of LEO was achieved by gentle finger rubbing while maintaining shell integrity.

#### 3.2.3. Alginate

Alginate is a natural polysaccharide derived from the cell walls of brown seaweed and is widely used in medical, pharmaceutical, food, and textile applications. In the food industry, alginate functions primarily as a thickener and binder. Industrially, refined alginate is obtained through two main processes—the calcium alginate method or the alginic acid method—both producing alginate with similar functional characteristics.

Alginate is regarded as one of the most suitable materials for encapsulation because it works well with various microencapsulation methods, including extrusion, layer-by-layer assembly, spray-drying, ionic gelation, and coacervation. However, certain limitations must be acknowledged, such as mechanical instability, environmental sensitivity, and batch-to-batch variability.

Among the available methods, extrusion followed by ionic gelation is one of the most commonly used. In this technique, alginate and the core material are co-extruded through a coaxial syringe into a calcium chloride (CaCl_2_) coagulation bath, where calcium ions serve as cross-linkers to form capsules or hydrogels [122].

**Figure 6:**
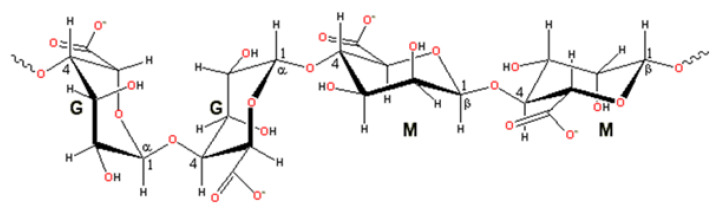
Alginate linear polysaccharide isomers (**M**) β-D-mannuronate, and (**G**) α-L-guluronate [124].

**Figure 7:**
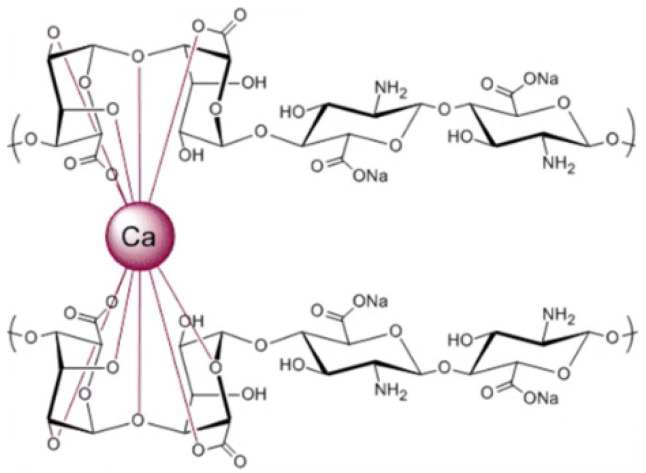
Diagram of the interaction of calcium and alginate G-G blocks structure. Adapted from Rinaudo [123].

**Figure 8:**
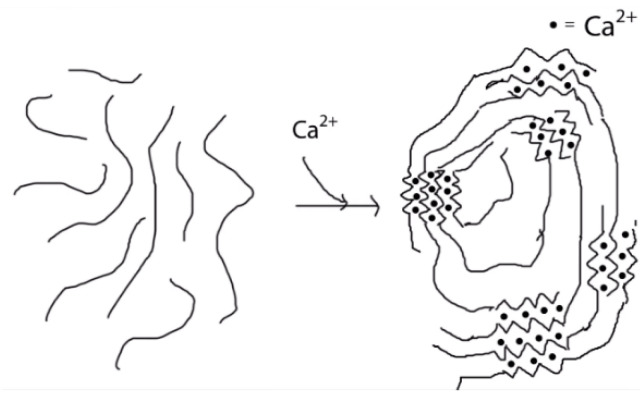
Schematic crosslinking representation of alginate in the presence of calcium counterions, completed with guluronic blocks. Adapted from Rinaudo [123].

Alginate is a linear anionic polysaccharide composed of β-D-mannuronate (M) and α-L-guluronate (G) residues linked through (1→4) glycosidic bonds arranged in blocks (M, G, and MG blocks) (Figure 6). Because of its anionic nature, calcium ions interact with the G-blocks, promoting ionic crosslinking and forming a stable gel structure (Figure 7 and Figure 8) [123].

Alginate also performs effectively in complex coacervation with chitosan. The carboxyl groups of alginates interact spontaneously with the protonated amino groups of chitosan, forming complexes through hydrogen bonding, electrostatic interactions, and dipole–dipole associations [123]. Other encapsulation techniques, such as spray-drying, extrusion, ionic gelation, and layer-by-layer assembly, are also possible but may present challenges, including excessive heat, long processing times, limited scalability, or the need for additional materials [123].

Several studies have demonstrated the effectiveness of alginate in encapsulating essential oils for textile applications. Specos et al. [90] encapsulated lavender oil in sodium alginate using an emulsion-extrusion method and reported improved fragrance durability during laundering. Liakos et al. [125] encapsulated cinnamon oil in alginate via spray drying and applied it to cotton fabrics, observing that the cinnamon oil’s antibacterial activity was retained even after multiple washing cycles.

In summary, alginate offers several advantages, including biocompatibility, mild processing conditions, and controlled-release behavior. However, its mechanical properties, environmental sensitivity, and variability may require optimization depending on the intended application.

#### 3.2.4. Cellulose-Based Microencapsulation Textiles

Cellulose is one of the most abundant natural polymers on Earth and constitutes the primary structural component of plant cell walls. It is obtained from sources such as wood, cotton, and agricultural residues. Chemically, cellulose is a linear polysaccharide composed of glucose units linked through β(1→4) glycosidic bonds, forming an unbranched chain [126].

Cellulose and its derivatives can be used to encapsulate essential oils (EOs). This approach is attractive because cellulose is biodegradable, biocompatible, and non-toxic, and can stabilize volatile compounds. Its structural features help to protect essential oils from environmental degradation and enable the controlled release profiles [126].

Several cellulose derivatives are used as shell materials for encapsulation, including hydroxypropyl methylcellulose (HPMC), microcrystalline cellulose (MCC), carboxymethyl cellulose (CMC), cellulose acetate, and nanocellulose. Although solvent evaporation is often involved in their processing, this method can introduce environmental concerns, prompting researchers to explore more sustainable alternatives.

Wunnoo et al. [127] encapsulated eucalyptus essential oil in HPMC microcapsules using an emulsion technique and applied them to cotton fabrics to impart antibacterial properties against *Escherichia coli* and *Staphylococcus aureus.* The treated fabrics retained significant antibacterial effectiveness after multiple washing cycles. Misni [128] encapsulated citronella essential oil in CMC using spray-drying to develop mosquito-repellent textiles; the fabrics exhibited effective repellency and moderate durability. Liakos et al. [129] encapsulated rosemary essential oil in cellulose acetate via electrospinning and deposited the fibers onto polyester fabrics. The resulting coatings demonstrated sustained release and strong antimicrobial activity.

#### 3.2.5. Cyclodextrin Monomolecular Inclusion Complex

Cyclodextrins (CDs) are widely used in the food, cosmetic, pharmaceutical, and chemical industries due to their ability to form inclusion complexes with a broad range of guest molecules [130]. Their application in the textile industry is less common but has gained attention in recent years, particularly for the stabilization and controlled release of essential oils (EOs).

Cyclodextrins are non-reducing, crystalline, water-soluble cyclic oligosaccharides. They consist of α-(1→4)-linked α-D-glucopyranose units arranged in a truncated cone shape, rather than a perfect cylinder, due to the chair conformation of the glucose units. The external surface of cyclodextrin is hydrophilic, while the inner cavity is hydrophobic, allowing the encapsulation of suitably sized non-polar molecules, including essential oils. Incorporation of EOs into the cyclodextrin cavity protects them from environmental degradation, improves stability, and enables controlled release when applied to textile substrates [130].

**Figure 9:**
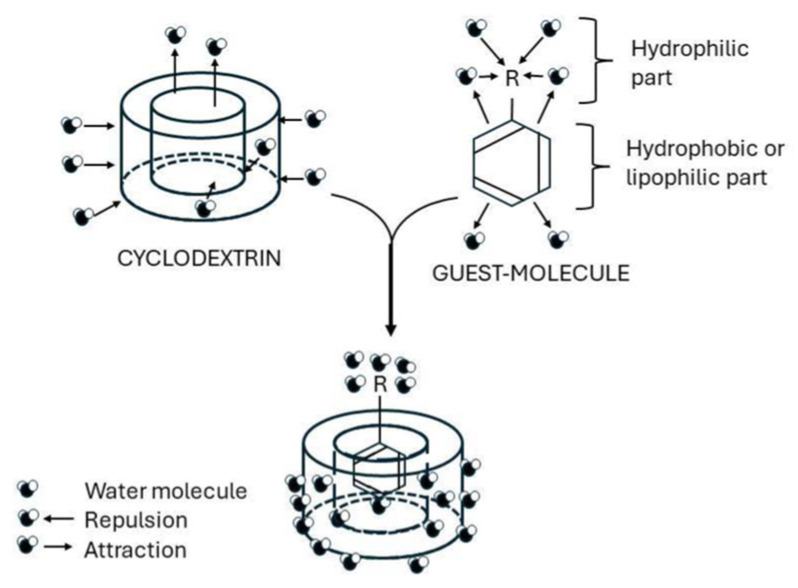
Cyclodextrin–guest molecule complex [130].

Lis Arias et al. [131] described several techniques for preparing EO–cyclodextrin inclusion complexes and applying them to textiles. The figure below (Figure 9) illustrates the formation of cyclodextrin–guest complexes.

The figure below will show cyclodextrin-guest complexes.

In terms of release kinetics, cyclodextrin compared with microcapsules results in difficulty because they exhibit a different release mechanism. While microcapsules typically rely on diffusion through a polymeric shell, shell rupture, or matrix erosion to achieve sustained delivery, clyclodextrin systems operate through a reversible host-guest equilibrium. For instance, the release of EOs from cyclodextrin cavities is primarily driven by desorption, moisture-triggered displacement, or competitive interactions with environmental molecules. Therefore, inclusion complexes provide moderate stabilization and relatively faster release profiles compared to core-shell microcapsules. Cyclodextrin systems are suitable for short-to medium-term fragrance or antimicrobial applications (Table 4), whereas polymeric microcapsules are more appropriate for prolonged, gradual release [132,133].

#### 3.2.6. Liposome

Liposomes are spherical vesicles composed of one or more phospholipid bilayers, structurally similar to biological cell membranes. Their bilayer architecture enables the encapsulation of both hydrophilic compounds (in the aqueous core) and hydrophobic compounds (within the lipid bilayer). This versatility allows liposomes to be applied in several fields, including drug delivery (DNA/RNA transport), cosmetic formulations, and even as auxiliaries in textile processes, such as wool dyeing [126].

Liposomes enhance the stability, bioavailability, and controlled release of active compounds. In textiles, essential oils can be incorporated into phospholipid bilayers to impart antimicrobial properties or provide skin-delivery functionalities.

The different interactions of liposomes toward natural and synthetic fibers can be attributed to differences in surface chemistry and polarity. Natural fibers such as CO, CL, WO, and S contain abundant polar functional groups (e.gl, hydroxyl, amino, and carboxyl groups), which promote hydrogen bonding and electrostatic interactions. These interactions enhance adhesion and reduce desorption from the textile surface. On the other hand, many synthetic fibers, particularly PES and PP, are predominantly hydrophobic and possess low surface energy with limited reactive functional groups [134,135]. Lis Arias et al. [131] demonstrated that liposomes interact well with natural fibers, exhibiting strong physicochemical affinity and low desorption, whereas their interaction with synthetic fibers is considerably weaker. These authors highlighted liposomes as effective vehicles for incorporating active compounds into textile substrates when bio-functional performance is desired. They compared two types of liposomes—internal wool lipids (IWL) and phosphatidylcholine (PC)—applied onto textile fibers.

IWL liposomes consist of cholesterol, free fatty acids, cholesterol sulfate, and ceramides, resembling components found in keratinized tissues like the stratum corneum or hair. PC liposomes consist of phosphatidylcholine molecules that form a bilayer, with hydrophilic head groups facing outward. This organization mimics biological membranes, making phosphatidylcholine-based liposomes biocompatible and suitable for controlled drug delivery.

The study concluded that liposomes are promising carriers for the application of active substances on textiles; however, the release behavior of liposomes and polymeric microcapsules differs fundamentally. Liposomes are phospholipid vesicles in which the release mechanism depends heavily on the physicochemical properties of the encapsulated compound. Hydrophilic actives tend to be retained within the aqueous core, while lipophilic actives are embedded within the lipid bilayer, resulting in different reservoir effects and release kinetics. Consequently, liposomal systems often exhibit relatively faster and more environmentally responsive release profiles. These systems typically provide higher loading capacity and more sustained reservoir effects. Therefore, liposomes are particularly suitable for short-term cosmetic or skin-contact applications, whereas polymeric microcapsules are more appropriate for prolonged, durable release [132,136].

## 4. Mechanical/Physical Process to Immobilize Essential Oils

Various processes can be used to create complexes that protect essential oils within a polymeric matrix, such as spray-drying, pan coating, extrusion, solvent evaporation, and vacuum deposition. In these methods, the wall material is mechanically deposited around the active core to safeguard it from environmental factors [137]. The final choice depends on factors like the equipment available at the textile plant, technical limitations, sustainability considerations, and the intended end use of the treated fabric.

### 4.1. Spray-Drying Microencapsulation

**Figure 10:**
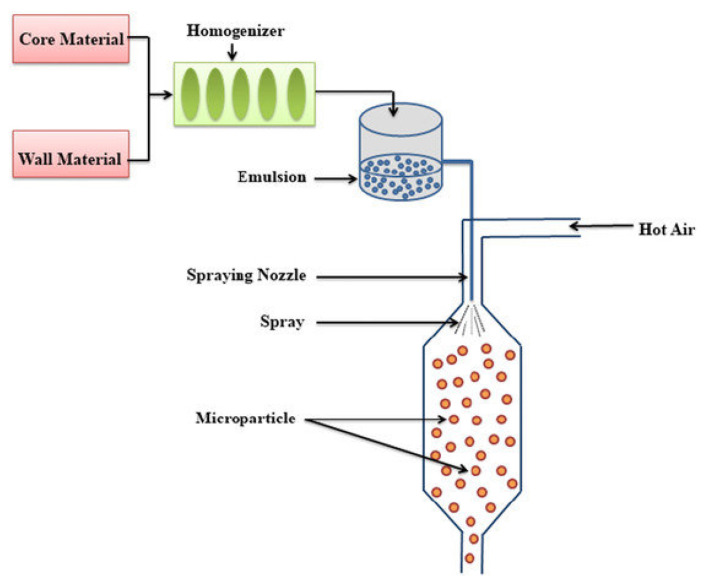
Schematic representation of spray-drying microencapsulation process [143].

Among microencapsulation methods, spray drying (Figure 10) has emerged as the most widely used technique for essential oil encapsulation due to its simplicity, reproducibility, scalability to industrial production, and relatively low operating cost [138]. In large-scale industrial settings, spray-drying provides stable manufacturing conditions and, compared to freeze-drying, reportedly costs 30–50 times less [139]. The process involves atomization of an oil-in-water emulsion followed by rapid solvent evaporation in a hot air stream, producing dry microcapsule powders with particle sizes typically in the micrometer range [140].

According to Misha [137], increasing the solid content of the wall material can enhance encapsulation efficiency. Spray-drying generally yields microcapsules with relatively rigid shells, though the choice of wall material remains critical and is often determined through trial-and-error optimization.

From a textile perspective, spray-drying offers several practical advantages (Table 4). The resulting microcapsules generally exhibit relatively rigid shells and good flowability, enabling their incorporation into finishing formations via a conventional pad–dry–cure process. Particle size distribution is particularly critical for textile applications, as excessively large particles can compromise fabric handle, while very fine powders may suffer from poor deposition efficiency without suitable binders. Consequently, binder selection and curing conditions strongly influence adhesion, wash durability, and long-term performance of fabrics. Wall material selection is limited by the requirement of water solubility or dispersibility, which favors polysaccharides and other hydrophilic biopolymers over certain synthetic polymers [141].

Despite its popularity, spray-drying presents several limitations. First, only wall materials with acceptable water solubility can be used. Second, the resulting microcapsules are fine powders that may require agglomeration to improve handling. Third, high temperatures during atomization can reduce oxidative stability and increase the loss of volatile compounds in essential oils [142].

### 4.2. Fluidized-Bed Coating

Fluidized-bed coating is regarded as one of the most effective and adaptable techniques for microencapsulating essential oils and other active compounds (Table 4). The process is relatively straightforward, achieves high encapsulation yields, and enables the coating to be performed in a single piece of equipment, making it very suitable for industrial use [141].

According to Bakry [143], the fluidized-bed coating process consists of two fundamental steps:

(a) Suspension of the core material

The core particles are suspended in an air stream with controlled temperature and humidity. The airflow must be stable and ascendant, from the bottom to the top. The purpose of this airflow is to fluidize the particles, enabling uniform exposure to the coating spray.

(b) Application of the coating material

A pump sprays the coating solution into the fluidized chamber, and the droplets adhere to the surface of the suspended core particles. Over time, repeated spraying and drying cycles generate a thin, uniform film around each particle, forming microcapsules.

As with any encapsulation technique, several parameters must be optimized to achieve high microencapsulation efficiency. The core material should possess an appropriate viscosity to facilitate pumping and atomization, and it must exhibit adequate thermal stability to withstand the hot air stream. Essential oils meet these requirements but must still be stabilized within a carrier matrix due to their volatility.

Particle morphology also affects coating performance. Spherical cores generally require less coating material to achieve uniform film formation, whereas irregularly shaped particles may lead to higher coating consumption. Particle size and size distribution are equally critical: a broad distribution makes it difficult to calibrate airflow conditions, often resulting in inconsistent coating thicknesses and reduced yield.

**Figure 11:**
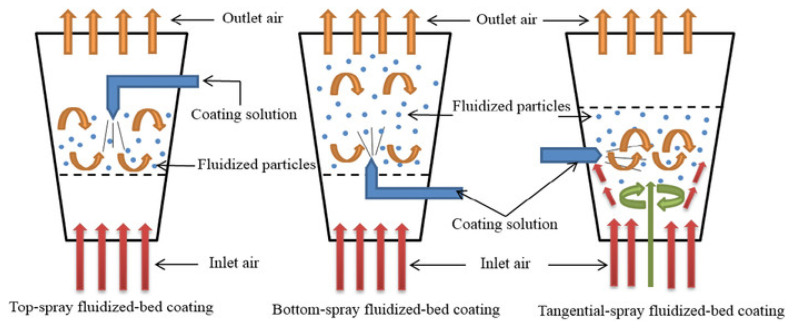
Schematic diagram to show top, bottom, and tangential fluidized-bed coating [143].

Fluidized-bed coating can be performed using several configurations, the most common being top spray, bottom spray (Wurster process), and tangential spray systems (Figure 11). While each configuration offers unique advantages, they all rely on the same principle—suspending core particles in a controlled airflow and depositing a continuous coating layer on their surfaces.

### 4.3. Extrusion

Extrusion is a well-known technique for microencapsulating essential oils; however, it is not commonly selected over other available methods (Table 4). This is mainly due to its high operational costs and the large size of the microcapsules produced, typically 500–2000 μm. Several processing parameters—including nozzle type and diameter, pump characteristics, flow rate, viscosity of the feed solution, surface tension, and the distance between the nozzle and the gelling bath—directly influence the final capsule morphology. These variables affect capsule size, sphericity, shell thickness, and structural integrity.

Despite these limitations, extrusion offers notable advantages. Essential oils such as cinnamon, clove, and thyme have shown improved encapsulation efficiency with this method, as extrusion significantly reduces evaporation and oxidation rates. As a result, the antifungal activity and storage stability of the encapsulated essential oils are enhanced when compared to spray-drying techniques [137].

**Figure 12:**
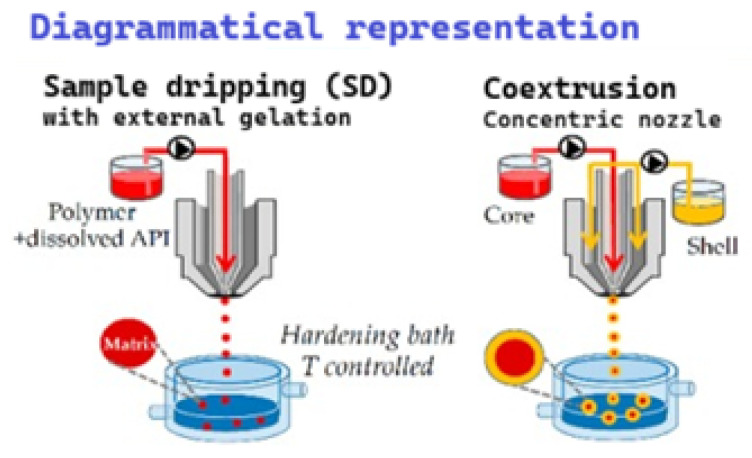
Schematic representation of droplet formation at an orifice initiates the preparation process [144].

Three main extrusion-based approaches are commonly used for encapsulating essential oils [137]:

(a) Concentric Nozzles (Figure 12)

This method uses two concentric tubes: the inner tube delivers the core material (essential oil), and the outer tube delivers the shell-forming polymer solution. Both streams are extruded through a single nozzle, forming droplets that fall into a hardening bath, where the shell polymer solidifies around the core.

The hardening bath typically contains either of the following:

- A non-solvent for the polymer (e.g., isopropanol);
- A crosslinking agent, which promotes rapid shell formation.

Droplet formation and final capsule properties depend on factors such as the following:

- Nozzle-to-bath distance;
- Viscosity of the polymer solution;
- Nozzle diameter;
- Surface tension;
- Concentration and type of crosslinker.

(b) Sample Dripping (External Gelation) (Figure 12)

In this method, a polymer solution containing dispersed essential oil is extruded through a nozzle into a hardening bath. The bath contains a non-solvent or a crosslinking agent that induces polymer gelation upon contact. Droplet formation is controlled by the nozzle flow and gravitational dripping mechanism.

As the droplets enter the bath, the polymer solidifies through external gelation, forming microparticles. Temperature and bath composition are strictly controlled to ensure proper crosslinking. After gelation, excess hardening liquid evaporates or is removed during drying, resulting in stabilized microcapsules.

### 4.4. Electrohydrodynamic Approach

The electrohydrodynamic approach is one of the most advanced and efficient techniques for encapsulating active compounds, including essential oils (Table 4). It relies on the interaction between high voltage and the surface tension forces of a polymeric solution. Although the underlying physics is complex, the overall process is conceptually straightforward and generally consists of three main stages.

In the first stage, a polymeric solution is prepared. This solution may contain the core and shell materials together or separately, depending on whether a uniaxial or coaxial configuration is selected. The viscosity and electrical conductivity of the solution are critical parameters that directly influence the success of the electrohydrodynamic process.

The second stage involves applying high voltage to the solution delivered through a syringe needle. Under the influence of the electric field, the liquid forms a Taylor cone and is ejected as a fine jet. Depending on the polymer solution’s rheological properties, this jet may elongate into ultrathin fibers or break up into solid particles.

The third stage consists of collecting the electrohydrodynamic products. The fibers or particles are deposited onto a grounded collector, which may be a stationary flat surface (such as a textile substrate) or a rotating drum, depending on the intended application [145].

The electrohydrodynamic process comprises two main techniques: electrospinning and electrospray. Their distinction lies primarily in the solution properties. The electrospinning technique requires higher polymer concentration and viscosity, enabling sufficient chain entanglement to form continuous nanofibers. In contrast, electrospray operates with low-viscosity, low-polymer-content solutions and produces solid micro- or nanoparticles.

A notable limitation is the poor electrospinnability of some biopolymers. For example, chitosan often exhibits excessively high viscosity and strong intra- and intermolecular interactions, making it difficult to electrospin for encapsulating essential oils. Electrospray, however, is more flexible and well-suited for producing microcapsules, particularly in uniaxial or coaxial configurations. In uniaxial systems, core and shell components are mixed in a single syringe; in coaxial systems, they are separated into two syringes, enabling distinct core–shell particle formation [145].

Electrospray offers several advantages, including low cost, room-temperature processing, atmospheric-pressure operation, and the potential to avoid crosslinking agents or post-treatment steps.

Electrospray is inherently slow, requires prolonged optimization, and makes it difficult to maintain consistent morphology and physicochemical properties. Moreover, encapsulating highly volatile compounds—such as essential oils—remains problematic. Electrical forces used in atomization can promote the volatilization of odorants alongside the solvent, thereby reducing encapsulation efficiency.

**Figure 13:**
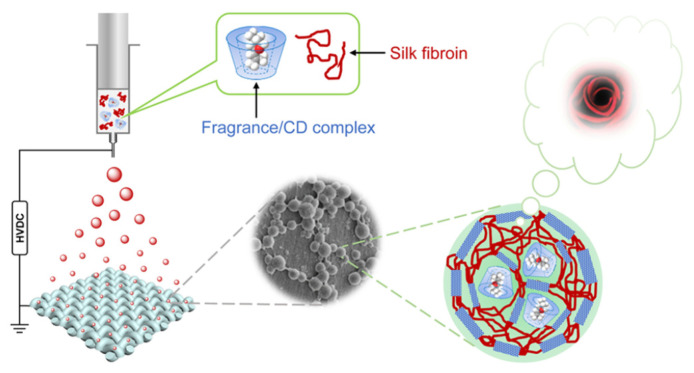
Schematic representation of bioactive aroma compounds in nanostructured matrices. [102].

To address some of these limitations, Long Ye and Zixie Li [102] (Figure 13) developed composite nanoparticles comprising regenerated silk fibroin (SF) and 2-hydroxypropyl-β-cyclodextrin (HP-β-CD) inclusion complexes loaded with fragrances. These nanoparticles were directly deposited onto silk fabrics during electrospray processing, demonstrating the feasibility of combining electrosprayed biopolymer nanocomposites with textile finishing.

### 4.5. Emulsification/Solvent Evaporation

The emulsification–solvent evaporation technique is one of the most widely used methods for microencapsulating essential oils. It relies on mixing two immiscible liquids—typically an organic phase containing the polymer and essential oil, and an aqueous phase containing a surfactant. The process begins by dissolving or dispersing the essential oil in the organic polymer solution. This organic phase is then emulsified into the aqueous phase containing a stabilizer, forming an oil-in-water (O/W) emulsion.

**Figure 14:**
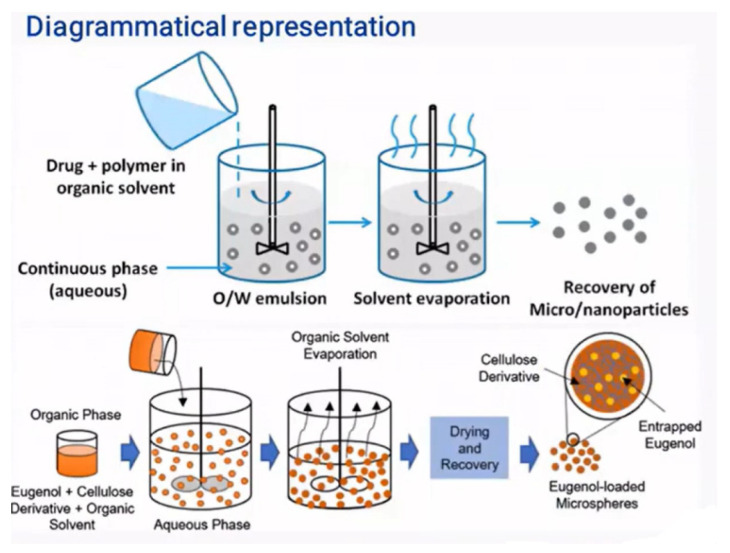
Solvent evaporation schematic representation to obtain microcapsules. Adapted from Wang and Simões [146,147].

Once the emulsion has been formed, the next step is to remove the organic solvent. This can occur either through solvent evaporation or solvent extraction, leading to polymer precipitation around the oil droplets and, ultimately, the formation of microcapsules (Figure 14) [83].

Microcapsules prepared via this method may be applied directly to textile substrates as aqueous dispersions or further processed into dry powders. In many cases, emulsification serves as an intermediate step in larger encapsulation processes. For instance, emulsions can be fed into spray-drying or freeze-drying systems, incorporated into extrusion methods, or used as templates for coacervation-based encapsulation [66].

A variety of emulsion systems can be used depending on the desired capsule morphology:

Oil-in-Water (O/W): oil droplets dispersed in water.
Water-in-Oil (W/O): water droplets dispersed in an oil phase.
Oil-in-Water-in-Oil (O/W/O).
Water-in-Oil-in-Water (W/O/W).

These multiple emulsions allow greater control over core–shell structures and release profiles. In a typical O/W system, oil droplets are stabilized by a surfactant that forms a protective interfacial layer, preventing coalescence and creating a kinetically stable dispersion. The stabilizer plays a critical role, as its lipophilic–hydrophilic balance governs droplet size, stability, and ultimately, the quality of the microcapsules produced [143].

### 4.6. Ultrasonication

Ultrasonication is an efficient technique that enhances microencapsulation processes, particularly for essential oils (Table 4). It is frequently used as a pretreatment to improve emulsion uniformity, reduce processing time, and generate liposomes or droplets with more homogeneous sizes—an important factor for obtaining consistent microcapsules in subsequent techniques such as coacervation [148].

**Figure 15:**
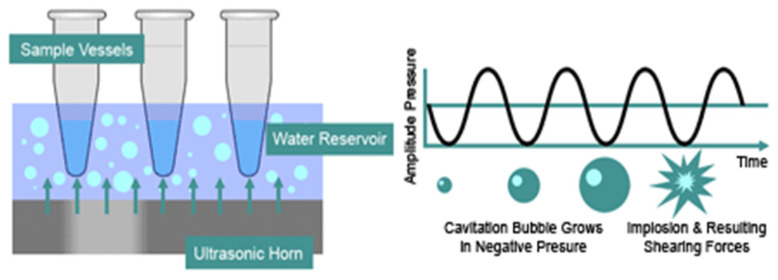
Ultrasonication schematic representation to get microcapsules. Adapted from Yetukuri [148].

This method relies on the application of high-frequency ultrasonic waves, typically 20 kHz to 500 MHz, at intensities above 1 W/cm^2^. When ultrasonic energy is introduced into a liquid medium, it induces acoustic cavitation—the formation, growth, and violent collapse of microbubbles. Cavitation produces localized high pressure and shear forces that temporarily disrupt lipid bilayers (e.g., liposomes), creating transient pores without destroying the vesicle structure (Figure 15).

These disruptions facilitate two possible outcomes:

- Negative-pressure cavitation, which allows external compounds—such as essential oils—to enter the liposome core.
- Positive-pressure cavitation, which can expel internal components (e.g., proteins) from within the vesicle.

Importantly, once the ultrasonic field is removed, the phospholipid bilayer reorganizes, allowing liposomes to regain structural integrity.

The ultrasonication process generally consists of two steps:

1. Sample positioning: Sample vessels are arranged in the ultrasonication chamber or within direct contact with the ultrasonic probe.
2. Sonication: The ultrasonic horn generates high-energy waves that disrupt the phospholipid chains, causing temporary membrane fractures and promoting molecular transport across the bilayer. During this process, water within oscillating bubbles undergoes hydrolysis, forming reactive H^+^ and OH^−^ radicals. These radicals may interact with amino acids or enzymes involved in maintaining membrane stability [148].

Overall, ultrasonication provides a rapid, efficient means of reducing droplet size and enhancing encapsulation uniformity, making it a valuable preparatory step for several microencapsulation techniques.

### 4.7. Cyclodextrin Co-Precipitation Inclusion Complexes Method

The cyclodextrin co-precipitation method is a straightforward and widely used technique for forming inclusion complexes between cyclodextrins (CDs) and essential oils (EO). The process relies on non-covalent interactions, primarily hydrophobic forces, that allow the guest molecule (EO) to enter the hydrophobic cavity of the CD.

The procedure generally involves preparing a saturated aqueous solution of cyclodextrin, followed by the dropwise addition of an ethanolic solution of essential oil under continuous stirring (typically for 1 h at 40–50 °C). After mixing, the solution is cooled to approximately 4 °C and stirred overnight to facilitate the formation and precipitation of the inclusion complexes.

The resulting precipitate is collected by vacuum filtration. A suitable organic solvent can be used to wash the precipitate, removing uncomplexed essential oil adhering to the CD surface. As described by Wadhwa et al. [149], this washing and filtration step ensures that only true inclusion complexes remain. The final product is then freeze-dried, yielding a dry powder of CD–EO complexes.

These dry inclusion complexes can be applied directly to textile substrates, particularly for odor-control applications. Cyclodextrin complexes with particle sizes below 12 µm are especially effective because their small size allows rapid dissolution upon contact with minimal moisture—often supplied naturally by human perspiration. As noted by Hedges [150], this rapid dissolution promotes the release of the entrapped essential oil, thereby enhancing odor neutralization and textile functionality.

From an industrial perspective, spray-drying and fluidized-bed coating remain the most scalable and commercially mature encapsulation technologies. Spray-drying offers high yield and relatively low cost per unit mass but entails significant thermal energy consumption, thereby contributing to its environmental footprint. On the other hand, extrusion and electrospray operate at milder temperatures; however, their low production throughput and longer processing times limit industrial scalability. Electrospray, although attractive for direct textile deposition and room-temperature processing, presents challenges related to solvent use, production rate, and large-scale reproducibility. Fluid-bed coating combines relatively high encapsulation efficiency with established industrial infrastructure, though it requires careful control of airflow and temperature. Therefore, the selection of an encapsulation method for textile applications must balance scalability, cost-efficiency, energy demand, and environmental impact rather than relying solely on encapsulation performance (Table 4).

## 5. Polymerization Methods to Encapsulate Essential Oils

The polymerization method is a relatively recent yet widely used technique for encapsulating essential oils, and it has become one of the most common approaches for producing microcapsules for functional textiles. In this method, polymerization occurs at the interface between the core material and the continuous phase in which the core is dispersed. Polymers such as urea–formaldehyde and melamine–formaldehyde are frequently used, as they form an insoluble polymeric shell around the core droplets, providing protection and stability to the encapsulated essential oil [137].

This technique is based on the reaction of monomer units at the interface separating the core and the continuous phase. The core can be either a liquid or a solid dispersed in a liquid medium. Similarly, the continuous phase may be a liquid or a gas, giving rise to different possible interfaces for polymer formation:

(a) When the core is in solid form, polymerization can occur at either the solid–liquid or solid–gas interface.
(b) When the core is dispersed as liquid droplets in the continuous phase, polymerization can occur at the liquid–liquid or liquid–gas interface.

Therefore, the polymerization-based encapsulation typically proceeds through two main procedural approaches.

### 5.1. In Situ Polymerization

In this method, the polymer shell is formed in the continuous phase and subsequently deposited onto the dispersed essential-oil droplets, thereby protecting the droplets from environmental factors. In in situ polymerization, no reactive species are added directly to the core material; instead, the polymerization reaction occurs exclusively in the continuous phase, and the resulting polymer deposits onto the surface of the dispersed globules [137].

**Figure 16:**
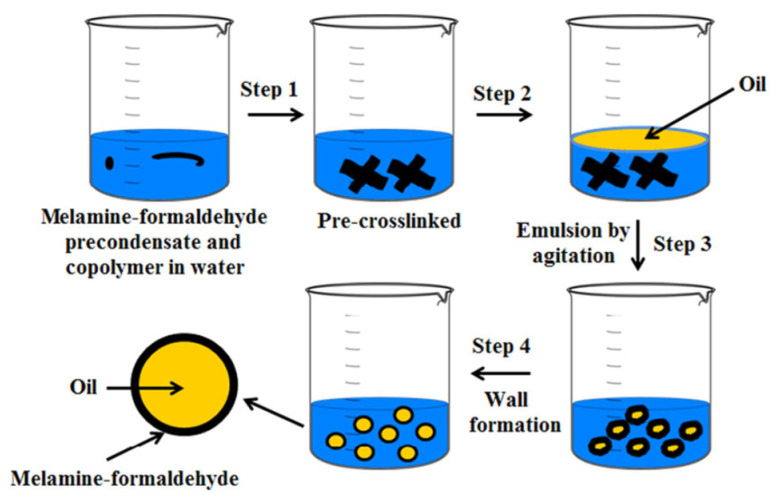
In situ polymerization schematic representation to get microcapsules [143].

The core particles are first dispersed, and the monomers (or prepolymer resin components) are dissolved in the continuous phase. When these monomers react—triggered by changes in pH, increased temperature, or the addition of a catalyst—an insoluble polymer is formed [137]. As polymerization proceeds, the newly formed polymer becomes less soluble, phase-separates from the continuous phase, and deposits onto the surfaces of the oil droplets. The deposited polymer then consolidates into a shell, completing the microencapsulation process (Figure 16). Microcapsules produced via this method typically range from 1 to 1000 µm in diameter [151].

Thyme, tea tree, and peppermint oils have been successfully encapsulated using melamine–formaldehyde resins [151]. For thyme oil, microencapsulation was performed via an emulsion-based procedure in which the oil was emulsified into an aqueous melamine–formaldehyde resin solution using sonication. The mixture was then stirred while the pH was adjusted to promote condensation between melamine and formaldehyde at the oil–water interface. This interfacial condensation led to the formation of a crosslinked polymer film surrounding the oil droplets, yielding stable melamine–formaldehyde microcapsules (Table 4).

### 5.2. Interfacial Polymerization

**Figure 17:**
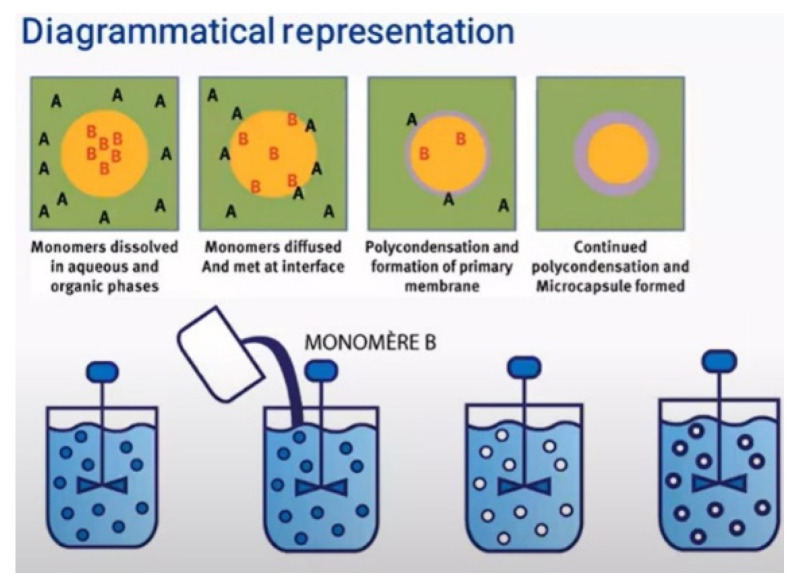
Schematic representation of interfacial polymerization for microcapsule formation. Adapted from standard models in the interfacial polymerization literature [152].

Interfacial polymerization occurs through four main steps. First, an oil-in-water emulsion is created, with two immiscible monomers in separate phases. The oil-soluble monomer (monomer A) dissolves in the dispersed organic phase along with the core material (essential oil), while the water-soluble monomer (B) dissolves in the continuous aqueous phase (Figure 17). An emulsifier is added to stabilize the emulsion. Polymerization then takes place at the boundary where the two monomers meet.

In the second step, monomers A and B diffuse toward the oil–water interface, where they come into contact and begin to react. In the third step, polycondensation is initiated by adjusting the pH, increasing the temperature, or adding a catalyst, resulting in the formation of a thin polymer shell around the dispersed droplets. Finally, the newly formed microcapsules are stabilized by isolation, washing, and drying. Microcapsules produced through this method typically range from a few micrometers to several hundred micrometers in diameter [151].

Scarfato et al. [153] developed polyurea microcapsules containing essential oils (lemon balm, lavender, sage, and thyme) for controlled-release applications using interfacial polymerization. In the study, the oil phase contained the essential oil and an oil-soluble isocyanate precursor (e.g., TDI) dissolved in pentyl acetate, and the aqueous phase contained water and 0.5 wt% Tween 80 as the emulsifier. Under agitation, the reaction between the isocyanate and a water-soluble amine at the interface produced a polyurea shell surrounding the oil core. The resulting microcapsules (10–15 µm) were characterized using FTIR, SEM, TGA, and HPLC. The results confirm successful shell formation and oil encapsulation with loadings of 20–25 wt%.

## 6. Physical/Chemical Methods for the Retention of Essential Oils

### 6.1. Layer-by-Layer

The layer-by-layer (LbL) technique is a widely used method for surface modification and for preparing functional nano- and microstructures. It involves the sequential adsorption of oppositely charged polyelectrolytes onto a core material, resulting in the controlled buildup of multilayer shells around the surface [154].

This technique was initially explored in biomedical applications to address issues such as rapid clearance of drug-loaded nanoparticles. Polyethylene glycol (PEG) was commonly used to functionalize nanoparticles; however, alternatives such as poly-L-glutamic acid (PGA) have been investigated to replace PEG in certain applications [154].

LbL assembly is cost-effective and does not require sophisticated equipment. It enables the design of delivery systems with tunable properties, and targeting can be achieved through passive, active, or physical mechanisms. Shell characteristics—such as thickness, composition, and permeability—can be adjusted by varying the number and type of layers. Furthermore, multilayer deposition provides enhanced protection of the encapsulated essential oil.

Zhang [155] fabricated microcapsules containing thyme essential oil using chitosan and alginate as the first and second layers. Their objective was to produce microcapsules with antibacterial properties and to study release behavior under different pH and temperature conditions. The characterization of the microcapsules was made using SEM and dynamic light scattering (DLS). The results showed that the antibacterial activity of thyme oil decreased with the increase in temperature, although inhibition improved when the oil was encapsulated. Antibacterial efficiency declined with increasing pH, while the microcapsules exhibited better stability under acidic or alkaline conditions. The microcapsules successfully inhibited the growth of *Staphylococcus aureus* in milk, indicating potential applications in both textile and pharmaceutical fields.

**Figure 18:**
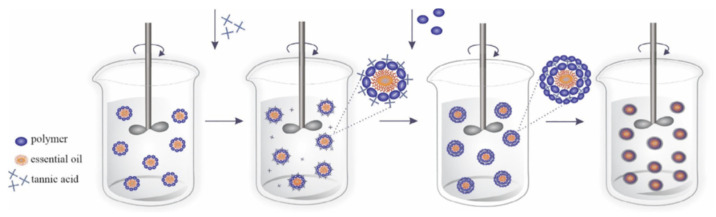
Layer-by-layer: Schematic diagram (color figure can be viewed at wileyonlinelibrary.com) [126].

In general, LbL assembly follows four main steps: (1) preparation of the core material (e.g., essential-oil microcapsules or droplets) using an emulsifier or surfactant; (2) adsorption of the first polyelectrolyte layer, often aided by a crosslinking agent; (3) deposition of a second polyelectrolyte with opposite charge, with additional crosslinking if needed; and (4) repetition of these cycles to achieve the desired number of layers surrounding the essential oil (Figure 18).

Despite its advantages, the LbL technique has several limitations when used for essential oils. Encapsulation yield may be low for highly volatile or reactive oils. Scalability can be challenging because the sequential deposition steps require precise control. Compatibility between the essential oil and the polyelectrolytes is not always guaranteed, and certain shell materials may be restricted by regulations in food, cosmetic, or pharmaceutical applications [154] (Table 4).

### 6.2. Coacervation

Phase coacervation is one of the oldest and most widely used microencapsulation techniques. It is generally divided into two categories: simple coacervation and complex coacervation. Coacervation involves phase separation of a polymer solution into two immiscible liquid phases: (a) a dense, polymer-rich coacervate phase and (b) a dilute equilibrium phase [151]. In complex coacervation, the separation results from electrostatic attraction between two oppositely charged biopolymers under controlled pH conditions.

(a) Simple coacervation

Simple coacervation is based on the phase separation of a single polymer solution. In this process, droplets of the coating polymer form around the dispersed core material after the polymer’s solubility is reduced. Phase separation can be induced by changes in pH, temperature, or by the addition of a non-solvent or inorganic salt. As the polymer becomes less soluble, it separates from the solution and forms polymer-rich droplets that deposit onto the core particles [138].

(b) Complex coacervation

**Figure 19:**
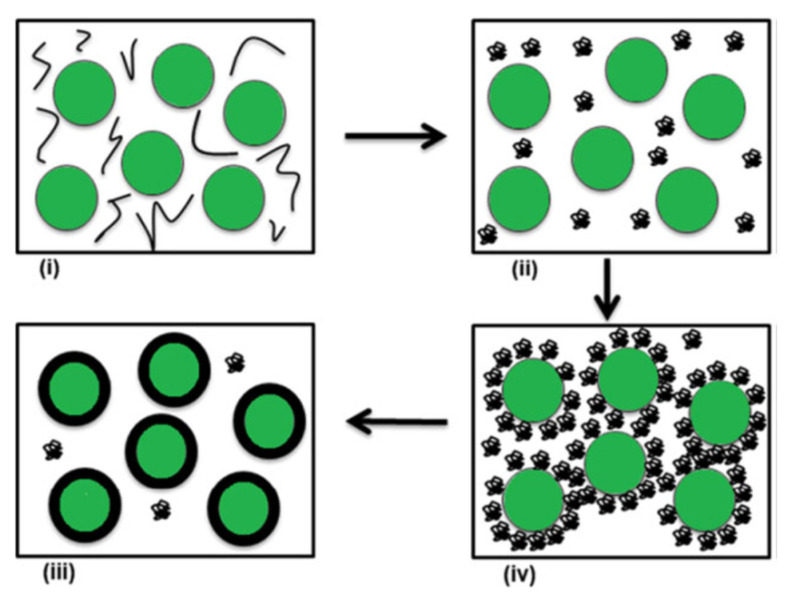
Example of complex coacervation involving (**i**) emulsification of oil in an aqueous solution containing two polymers, (**ii**) induction of coacervation by lowering the pH, (**iii**) deposition of the polymers onto the oil droplets, and (**iv**) shell hardening by crosslinking [143].

Complex coacervation occurs when two polymers with opposite electrical character interact in solution, forming a coacervate phase. A schematic representation is shown in Figure 19. Under appropriate conditions—typically by lowering the pH below the isoelectric point of one polymer—the oppositely charged molecules associate and deposit onto the surface of the dispersed oil droplets.

The technique generally involves four steps under continuous stirring:

Dispersion of the active substance (essential oil) in a solution containing a surface-active hydrocolloid.

Precipitation of the hydrocolloid onto the dispersed droplets by reducing its solubility (e.g., pH change, non-solvent addition, temperature shift, or electrolyte).

Addition of a second hydrocolloid with opposite charge to induce polymer–polymer complexation in the case of complex coacervation.

Stabilization and hardening of the microcapsules through crosslinking.

The shell formed through complex coacervation is critical, as it must effectively protect the encapsulated essential oil. Polymers such as Arabic gum (negatively charged) with chitosan or gelatin (positively charged) are commonly used, with pH adjustment enabling their interaction [137]. Crosslinking is often introduced to improve shell strength. For example, Rojas-Moreno [156] studied how different crosslinkers affected the encapsulation efficiency (EE) of orange essential oil using whey protein isolate (WPI) and chitosan. Tannic acid, sodium tripolyphosphate, oxidized tannic acid, and transglutaminase were evaluated. The highest EE was obtained when tannic acid was used as the crosslinking agent.

Polyvinyl alcohol (PVA) can also serve as a wall-forming material in simple or complex coacervation due to its hydrophilicity and ease of processing. PVA may be further crosslinked with glutaraldehyde to form a hydrogel when higher mechanical stability is required [157].

There are advantages and limitations associated with simple and complex coacervation (Table 4).

Simple coacervation is generally less expensive because it relies on inorganic salts to induce phase separation. In contrast, complex coacervation is more sensitive to small pH changes and typically employs more costly hydrocolloids.

Complex coacervation provides better-controlled release properties and greater shell stability, particularly when multilayer coatings are formed.

In summary, coacervation offers significant advantages over other encapsulation techniques. It is economical, reproducible, and scalable, and can produce microcapsules without requiring organic solvents—an essential feature for environmentally friendly and food-grade applications [155].

**Table 4:** Comparative assessment of immobilization strategies for essential oils in textile substrates.

Method	Advantages	Limitations	Textile Relevance	Key Ref.
Complex coacervation	High encapsulation efficiency for volatile terpenes, good protection	Requires crosslinkers (glutaraldehyde); sensitive to pH	Widely applied in antimicrobial via pad-dry-cure	Xiao [158]
β-cyclodextrin	Molecular stabilization of small EO molecules and wash durability	Lower loading capacity	Fragrance and moderate antimicrobial	Dai [159]
Spray-drying	Low-cost; scalable; protects EOs from evaporation during process	Lower encapsulation efficiency, weaker adhesion	Disposable textiles	Rosemberg [160]
Fluid-bed coating	Uniform EO-loaded coating; scalable; suitable for industrial textile finishing	Equipment cost	Textile microcapsules finishing via pad-dry-cure.	Srivastava [161]
Extrusion (melt extrusion)	Solvent-free; continuous processing; good encapsulation in thermoplastic matrices	High temperature may degrade volatile EO components	Functional synthetic fibers (e.g., PES, PP, PA)	Pargai [162]
Electrohydrodynamic techniques	Nanofiber encapsulation, high surface area, controlled release	Low productivity; scaling challenges	Advanced medical textiles and wound dressing	Rivero [163]
Ultrasonication	Produces stable EO nanoemulsions; improves emulsion stability; enhances encapsulation uniformity	Limited long-term stability without additional crosslinking	Pre-encapsulation step for textile coating system	Puntipa [164]
In situ polymerization	Strong mechanical stability	Synthetic polymer: possible toxicity concerns	Long-term durability	Patil [165]
Interfacial polymerization	Strong, dense polymer shells; high mechanical durability.	Synthetic monomers; potential toxicity concerns; regulatory issues	Long-term antimicrobial textile requires durability	Song [166]
Solvent-evaporation	Controlled release tuning	Solvent residues; processing complexity	Functional medical textiles.	Tiwari [167]
Layer-by-Layer	Precise nanoscale control; compatible with textiles	Multi-step process; cost	High-performance biomedical and smart textiles	Fan [168]

## 7. Methods to Apply Essential Oils on the Textile Substrate

There are two well-known approaches to application:

− Direct Application of essential oil on the textile surface, where the EO is not immobilized [26].
− Indirect Application (such as carriers) of essential oil on the textile surface, where the chemical compounds from EO are immobilized. They can protect chemical compounds from Essential oils against harsh environmental conditions. These processes can be divided into chemical, mechanical/physical, chemical/physical, and emulsion.

This review will provide information on these two approaches mentioned above.

Table 2 summarizes the most common methods for functionalizing textiles.

### 7.1. Application of Essential Oil Through the Direct Approach (Not Immobilized)

In this methodology, essential oils are applied to the textile surface without protection. Consequently, the chemical constituents are exposed to the environment, making them unstable and prone to rapid evaporation. Essential oils are volatile compounds that evaporate when exposed to air and are sensitive to sunlight. Therefore, this approach shows low yield because the release profile cannot be controlled accurately, and certain oils may damage fabrics, causing stains or skin irritation depending on the quantity applied [26,169].

The direct application of essential oils onto textile surfaces can be performed using several methods [170,171]:

1. Infusion method: A few drops of EO are applied onto a cotton ball and placed inside a container with the fabric to allow gradual fragrance absorption. This method is more suitable for natural fibers.
2. Dropped method: A few drops of EO are applied directly onto the textile surface to achieve localized application. Care must be taken to prevent staining or damage to the fabric.
3. Ironing method: A few drops of EO are added to water and sprayed onto the fabric prior to ironing. Heat activates the fragrance and enhances absorption.
4. Roll-on method: EO is applied directly onto the textile using a roll-on applicator. This method is suitable for small, targeted areas (e.g., collars and cuffs) but may cause fabric damage.
5. Immersion method: EO is added to fabric softeners or detergents during laundering, allowing fragrance infusion into the fabric.
6. Spray method: EO diluted in water is sprayed onto technical fabrics such as curtains, upholstery, or carpets.

Sadaf [170] employed a mixed approach using both roll-on and ironing methods. Lavender, rosemary, and tea tree oils were applied directly to cotton and polyester fabrics to provide fragrance and potential health benefits. The pad-dry-cure method was used, followed by heat pressing. Baking soda (NaHCO_3_) was recommended as a binder to improve scent durability.

Srivastava [171] compared aroma-retention properties in silk, cotton, and wool using the immersion method. Fabrics were immersed in solutions containing 10–50% EO for 24 h, squeezed, dried, and cured. Wool showed the highest aroma retention according to olfactometry.

**Figure 20:**
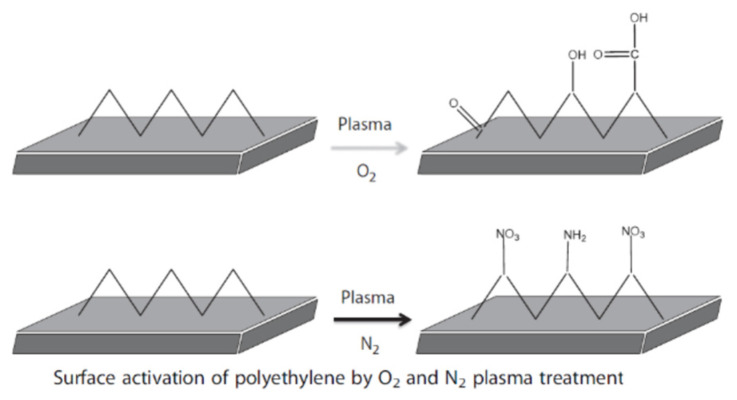
Textile after plasma treatment [172].

Reda [172] applied lavender, thyme, and vetiver EOs by the exhaustion method for medical textiles. Fabrics were treated for 20 min at 40 °C. The antimicrobial and healing properties of these oils were utilized. Plasma treatment (Figure 20) was used to enhance EO uptake and functional properties.

On the other hand, several techniques—including padding, exhaustion, electrospinning, spraying, and grafting—are used to apply the immobilized EO on fabrics. These methods generally yield higher quality because the antibacterial, antimicrobial, insect-repellent, and fragrance properties of EO are preserved for longer periods, and the diffusion of active compounds can be controlled.

However, determining the most suitable application method is challenging for two reasons: it depends on the EO complex’s compatibility with the fabric, and it is constrained by the machinery available in the manufacturing process. Some of these techniques are classified as chemical finishes [126] because they resemble dyeing processes, where EO application is performed similarly.

Other techniques apply immobilized EO through combined chemical/physical processes such as exhaust (batch processing after dyeing), padding and curing (immersion followed by squeezing and heat treatment), spraying, printing, foam application, or vapor deposition. EO-based finishes may also be added to the spinning bath before manufacturing man-made fibers.

The choice of method depends largely on the fiber. If the finishing chemical has a strong affinity for the fabric, the exhaust bath after dyeing is suitable. If the chemical has a lower affinity, continuous processes—such as immersion or mechanical application—are recommended to improve uptake and durability [51].

### 7.2. Application of Essential Oil Through the Indirect Approach (Immobilized Essential Oils)

#### 7.2.1. Padding

According to Choudhury and Giamberini [13,14], padding is one of the most widely used techniques in the textile industry for applying immobilized essential oils (Table 5). This method can provide durable finishes and multifunctional properties such as repellent of insects, antimicrobial activity, fragrance delivery, and aromatherapy effects [173,174,175,176,177].

**Figure 21:**
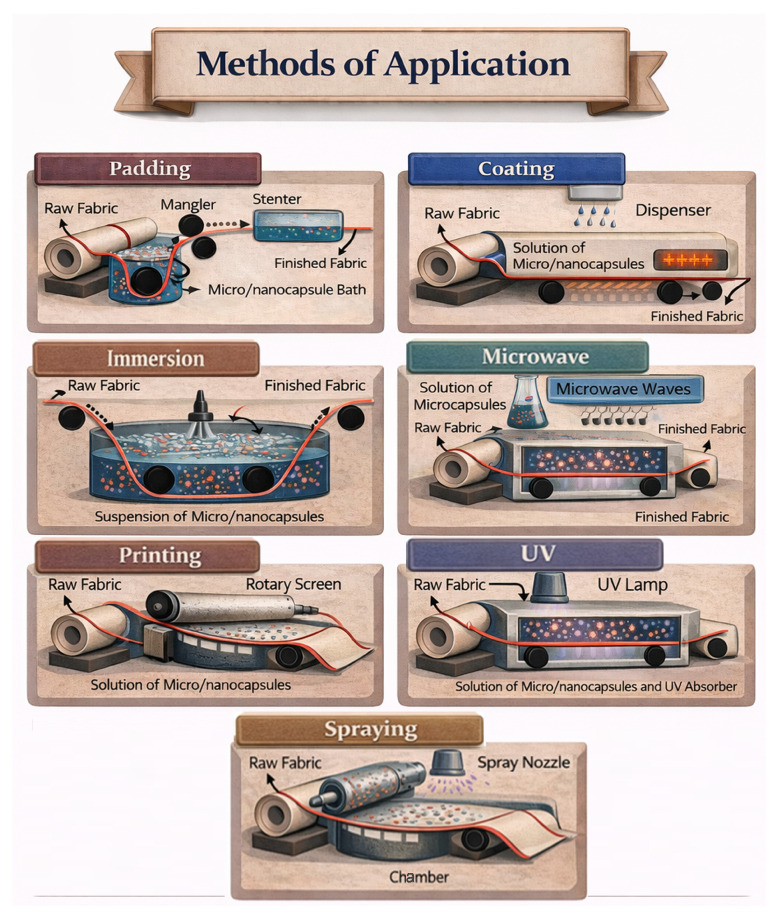
Various methods for the application of micro/nanocapsules on textiles.

Padding is the first step in a sequence of operations used to introduce microcapsules or complexes onto textile substrates [126]. The primary objective is to uniformly distribute the finishing agent before fixation. Figure 21 summarizes the sequence of operations involved. In this technique, fabrics are continuously passed through a bath containing the finishing agent (immobilized EO). A pair of rollers squeezes the fabric to control the pick-up (the percentage of liquid retained by the fabric). The fabric is then dried, often at elevated temperatures. Drying is important because higher temperatures promote the fixation of the finishing agent. Moisture is also removed during this step. Several methods—UV radiation, infrared radiation, or microwave drying—may be used for the drying stage.

Although padding is widely used to achieve homogeneous application of finishing agents, it may reduce certain comfort properties, such as softness and air permeability [178].

Stan [17] achieved good results using the padding method (Table 5) to obtain a homogeneous distribution of Sage and Rose EO microcapsules on textile substrates for dermal applications. High temperatures during the drying stage allow the use of a commercial acrylate-based binder, extending microcapsule durability up to five washing cycles and 1000 abrasion cycles. In vitro biocompatibility tests on human skin cells confirmed the absence of cytotoxicity after short-term exposure.

Kert and Tavcer [179] applied fragrance EO microcapsules by padding after exposing the fabric to low-pressure nitrogen and oxygen plasma to enhance adsorption and adhesion. The authors reported that padding provided a more uniform application than other methods. Plasma treatment improved the wicking properties of cotton, and O_2_ plasma caused slight fiber etching, which increased tensile strength.

#### 7.2.2. Spraying Method

Spraying is another method for applying finishing agents, such as immobilized EO, to textile surfaces. In this technique, the fabric is placed in a closed environment, and the finishing agent is sprayed as a fine mist across the fabric surface. Several process parameters can be controlled, including the add-on level, spray rate, rotation time, and processing duration. Crosslinking agents or binders may be incorporated into the spray formulation. After spraying, the fabric is typically pressed and cured in a hot-air chamber [13,168,180,181,182].

Ye and Li [102] reported an innovative approach using electrospraying (Table 5) to immobilize EO and fabricate silk nanocomposites. They prepared nanocomposites using an all-aqueous solution of fragrance/2-hydroxypropyl-β-cyclodextrin inclusion complexes and regenerated silk fibroin, achieving aroma-encapsulation efficiencies greater than 90%. Ye and Li [102] explored two objectives: first, to analyze the release mechanism of nano-encapsulated fragrance, and second, to eliminate the need for a separate finishing step by incorporating immobilized fragrance directly during fabrication. The results showed that EO/HP/β-CD silk nanocomposites preserved volatile compounds more effectively than the simple inclusion complexes and exhibited near-zero-order release kinetics with a slow-release rate upon alcohol treatment. Finally, the fragrance-carrying nanoparticles were successfully applied to silk fabrics during the electrospraying process.

#### 7.2.3. Immersion/Exhaustion

Immersion/exhaustion is a commonly used batch method for applying immobilized EO to textile substrates. This process is particularly effective for treatments that require prolonged and controlled release. When deep penetration and improved adhesion of immobilized EO are needed, the immersion/exhaustion method may be suitable [13,14,183].

Although related to padding as a wet-application technique, exhaustion differs in eliminating the use of squeeze rollers. The fabric is immersed in a bath containing the finishing agent (immobilized EO) under controlled conditions of temperature and bath composition (salts, pH, surfactants, etc.) and kept in the bath for a defined period. Afterward, excess liquid may be removed by hydro-extraction or squeezing.

Bonet [184] applied melamine–formaldehyde microcapsules containing lavender EO to compare exhaustion and padding as finishing processes (Table 5). Cotton fabrics (100% CO) were immersed in an exhaustion bath containing lavender microcapsules and a resin binder. The study showed that a significant proportion of microcapsules was lost to the wastewater. Although the bath was reused, the microcapsules had swollen and ruptured due to prolonged exposure, rendering the bath unsuitable for reuse. The padding is generally more feasible for microcapsule application because the mechanical pressure from the rollers enhances capsule deposition on the fabric.

Ali [183] synthesized chitosan nanoparticles (CSN) using sodium tripolyphosphate (TPP) and subsequently loaded them with silver ions to produce Ag-CSN. These nanoparticles were applied to PES fabrics via exhaustion to impart antibacterial properties. The fabrics were immersed in a 0.2% (*w*/*v*) chitosan solution, CSN dispersion, and silver-loaded CSN dispersion for 45 min at 60 °C. The release of Ag^+^ from Ag-CSN-finished PES was confirmed by antibacterial testing, which showed a distinct zone of inhibition.

#### 7.2.4. Grafting

Grafting is a method in which polymer chains are chemically attached to the backbone of another polymer or directly onto a material surface, such as a textile fabric [3,4]. This technique can improve the durability of immobilized EO by facilitating the formation of chemical bonds between the finishing material and the textile substrate.

Grafting is a flexible technique and can be combined with various immobilization approaches, including microcapsules, polymer complexes, and cyclodextrin inclusion systems [185,186,187]. The polymer matrix protecting the EO may be covalently attached to the fiber surface through polyfunctional crosslinking agents. Dimethylol dihydroxyethylene urea, 1,2,3,4-butane tetracarboxylic acid, and citric acid are among the most commonly used crosslinkers. Grafting is frequently applied to textile substrates to tailor properties such as hydrophilicity and surface reactivity.

Alonso and Gimeno [187] successfully grafted chitosan-based microcapsules containing grapefruit seed EO onto cellulose fibers to develop biofunctional textiles with antibacterial activity (Table 5). Cellulose was first exposed to UV irradiation to create reactive sites for subsequent grafting, after which the substrate was functionalized using an aqueous chitosan microcapsule emulsion containing EO. SEM and gas chromatography–mass spectrometry (GC–MS) analysis confirmed successful attachment. The treated materials showed 100% inhibition of *Escherichia coli* and *Staphylococcus epidermidis* for up to 48 h, attributed to the presence of chitosan. Additionally, the fragrance of grapefruit seed EO was retained for up to six months.

Khanna [186] used monochlorotriazinyl β-cyclodextrin (MCT β-CD) complexed with clove, eucalyptus, and peppermint oils to evaluate release behavior from functionalized cotton (Table 5). The grafting process was optimized using response surface methodology with MCT β-CD concentration, pH, and curing temperature as independent variables. The study demonstrated that EO retention in MCT β-CD inclusion complexes—both before and after washing—was significantly higher than that of uncomplexed EO applied to cotton.

#### 7.2.5. Coating Method/Screen Printed

The coating method for applying immobilized EO to textile surfaces involves preparing a solution containing dispersed microcapsules of EO, a binder, and a surfactant. The binder forms a polymeric film on the fabric surface that helps anchor the immobilized EO and supports controlled release during use [13,14].

Several methods can be used to apply the coating mixture, including knife-over-roll, screen printing, and spraying. The objective is to distribute the immobilized EO uniformly across the fabric. After application, the coated fabric is dried to remove solvent and cured (when required) to improve adhesion.

Golja and Tavcer [89] investigated the applicability of the screen-printing method (Table 5) for the microencapsulated EO onto cotton fabrics to impart fragrance, flame-retardancy, and antimicrobial properties. Microcapsules were prepared via in situ polymerization using melamine–formaldehyde as the wall material and three different EO core materials. The optimal number of microcapsules in the printing paste was evaluated to achieve durable fragrance, antimicrobial activity, and flame-retardant properties. Mechanical properties of the treated fabrics were analyzed before and after washing. Through iterative optimization, the authors identified the appropriate paste concentration for each microcapsule type. They concluded that the screen-printing method is suitable for applying melamine–formaldehyde microcapsules using synthetic swelling thickeners and polymeric binders (pigment system).

## 8. The Market for Immobilized Essential Oils Applied to the Surface of Textiles

**Figure 22:**
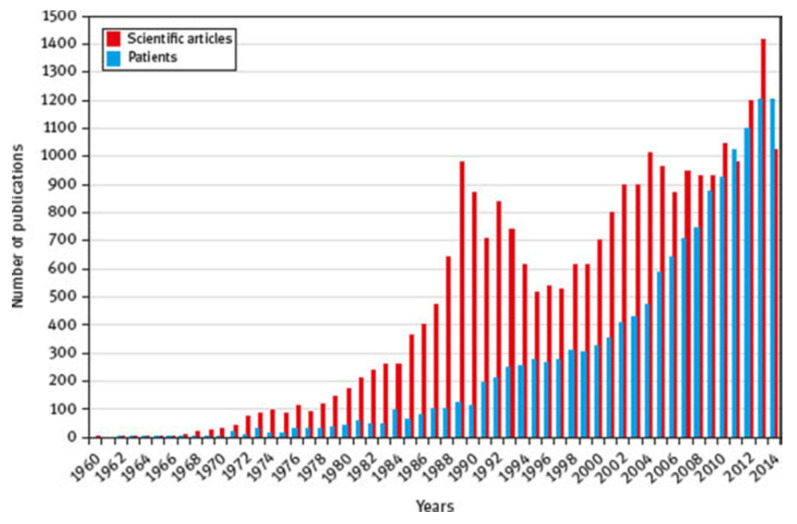
Trends in scientific articles vs. patent documents on microencapsulation. Web of Science [188], advanced search: TS = (microcapsule* OR microencapsulat*) AND TS = (textile* OR cloth OR fabric OR garment*).

As discussed previously, EO compounds can be immobilized using several techniques, including micro- and nanoencapsulation, liposome formation, and cyclodextrin inclusion complexes. Among these, encapsulation technologies (micro- and nanoencapsulation) are the most widely used in the textile industry for protecting volatile EO compounds (Figure 22). Encapsulation has grown substantially and is now used in a broad range of industrial applications beyond textiles.

**Table 5:** Application of microcapsules on the textile surface to get bio-functionalities with emphasis on antibacterial properties.

Encapsulation Method	Shell Material	Crosslinking	Core Material	Preparation Method	Functional Textile	Ref. No.
Spray-drying	Chitosan	---	Cinnamon	Pad-dry method	Antioxidant, antibacterial, and mosquito repellent	Singh, N. [80]
Spray-drying	Acacia gum	---	Citronella	Exhaustion method	Skin reduces irritation	Yingngam [36]
Not informed	Melamylformaldehyde	Acrylic	Lavender	Exhaustion method	Wash durability	Bonet [184]
Simple coacervation	Chitosan	---	Citrus	Exhaustion method	Antimicrobial woven cotton fabrics	Julaeha, E. [92]
Simple coacervation	Gelatin	---	Eucalyptus	Pad-dry method	Antimicrobial (reduces asthma and allergy)	Kim, J. [60]
Simple coacervation	Cellulose acetate and Chitosan	---	Eucalyptus	NA	Antimicrobial activity and Wound dressing	Elbhnsawi, N [85]
Simple coacervation	Xanthan Gum and Gelatin	---	Lavender oil	NA	Skincare textile	Danila, A. [189]
Complex coacervation	Chitosan and Arabic gum	---	Lime oil	Exhaustion method	Antibacterial activity	Wijesirigunawardana [190]
Emulsion	Arabic gum and Gelatin	---	Propolis	Padding	Antibacterial activity	Yaman, T. [191]
Interfacial polymerization	Melamilformaldehyde	Acrylate-based	Sage and Rose	Padding	Antibacterial activity	Stan [17]
Emulsion	Chitosan/Sodium Alginate	---	Lemmon grass	Grafting	Clinical treatment of atopic dermatitis	Chi, P. [192]
Complex coacervation	Chitosan and Arabic gum	Tannic acid	Limonene and vanillin	Grafting	Antibacterial cotton textiles	Sharkawy, IP. [193]
Simple coacervation	β-cyclodextrin	Resin	Citronella	Grafting	Insect repellent in textiles	Bouaziz, A. [185]
Co-precipitation	β-cyclodextrin	---	Calamansi	Pad-dry-cure	Antibacterial properties	Farouk [194]
Co-precipitation	β-cyclodextrin	---	Citronella oil	Pad-dry-cure Crosslinking	Repellent Agents	Lis [82]
Co-precipitation	MCT-βCD	---	Eucalyptus, peppermint, lavender	Textiles were treated with an ethanol (ester binding) solution by spray	Fragrance	Khanna [195]
Co-precipitation	β-cyclodextrin	---	Citronella	Grafting (covalent interaction with chemical groups from WO)	Repellent Agents	Bezerra [196]
Coacervation	Alginate/Chitosan	---	Lime peel EO	Pad-dry with binder	Anti-bacterial	Indriyani [197]
Ionic Gelation	Alginate	---	Neem Oil	Coating	Anti-bacterial efficacy	Khan [198]
Microemulsion	Alginate	---	Pepper Mint	Spray-drying	Antibacterial	Ghayempour [199]
Electrospraying	HP-β-cyclodextrin	---	Fragrance	Spray-drying	Aroma	Long Ye [102]
Solvent diffusion	Alginate	---	Coconut Oil	Printing	Antibacterial and aroma finishes	Lopez [200]
Emulsion-based encapsulation	Alginate	---	Various	Not specified	Anti-microbial and anti-fungal properties	Liakos [125]
Pickering emulsion	Chitosan	---	Cinnamon	----	Anti-bacterial system	Yang [78]
Complex Coacervation	Chitosan/Gelatin	---	Cinnamon	Padding	Anti-bacterial	Singh [80]
Complex Coacervation	Gelatin/Arabic-Gum	---	Fragrance	Padding and coating	Fragrance	Miro Specos [90]
Coacervation	Gelatin/Arabic-Gum	---	Fragrant Vetier	Pad-dry-cure	Fragrance	Rukhaya [201]
Emulsion solvent diffusion	Ethyl cellulose/silica hydrid	---	Lavender	Coating	High UV-resistance and durable aroma	Chen [202]
Phase separation	Ethyl cellulose	---	Rosemary and Lavender	Different techniques: Padding, spraying, impregnation, and exhaust	Durable fragrances, antibacterial agents, skin softeners, phase change material	Badulescu [203]
Emulsion	Chitosan	---	Grapefruit seed	Exhaustion	Anti-bacterial	Alonso [187]
Inclusion Complex	MCT-β-cyclodextrin	---	eucalyptus	Graft	Chemical compounds concentration fabric	Khanna [186]
in-situ polymerization	Melamilformaldehyde	---	Rosemary, Sage, Lavender	Print screen	Optimal microcapsules conc. In the printing paste to textile functionalization: flame retardant/antibacterial/fragrance	Golja [89]

Consumption of bio-functional textiles has increased, as consumers now seek fabrics that combine traditional functions (protection, comfort, aesthetics) with additional benefits and environmental sustainability. Such textiles may incorporate antimicrobial agents, insect repellents, UV protection, fragrances, and even cosmetic or medicinal actives. As a result, the market has recognized that companies may gain a competitive advantage by investing in innovation.

Textile manufacturers involved in spinning, weaving, and finishing have been encouraged to adopt new technologies that provide enhanced functionalities. Research related to immobilized active compounds—microencapsulation, inclusion complexes, and nanoparticles—has increased significantly. Applications include aromatherapy, antifungal and antibacterial textiles, medical fabrics, insect-repellent clothing, and cosmetic textiles.

**Figure 23:**
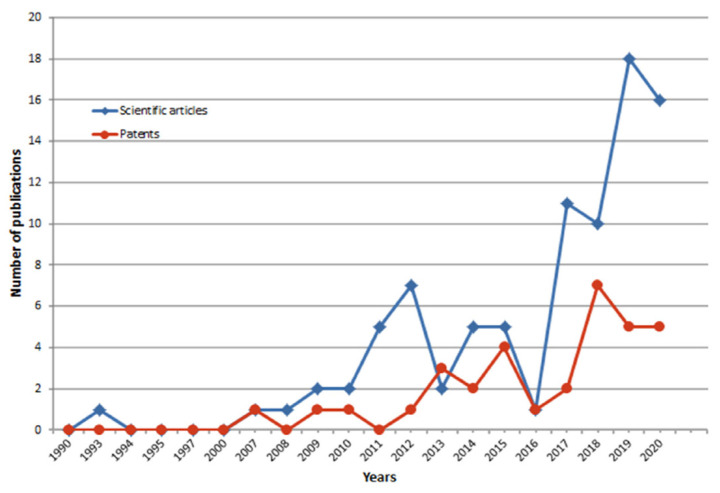
Trends in scientific articles vs. patent documents on microencapsulation for textiles. Web of Science [188], advanced search: TS = (microcapsule* OR microencapsulat*) AND TS = (textile* OR cloth OR fabric OR garment*).

Figure 23 shows a clear trend of increased publications and patents over recent years. Academic research has explored diverse applications of EO microcapsules, while industrial patenting suggests a growing interest in commercializing these technologies.

Research into EO immobilization on textiles began in the early 1980s. Initial efforts focused on encapsulating fragrances for textile finishing. Two main challenges emerged: (1) achieving controlled aroma release and (2) identifying crosslinkers that provided durable fixation after multiple wash cycles.

Sunidth Mehta [15] published an extensive review of aromatherapy applications on textiles, including over 300 publications from the past ten years. They noted substantial progress but also identified a gap: many aromatherapy studies assess the therapeutic effect of EOs in conventional settings (e.g., massage) rather than evaluating immobilized EO delivery via textiles. Existing textile studies often emphasize encapsulation methods and release mechanisms, but few assess the drug delivery effects to human skin.

EOs have also been immobilized to develop insect-repellent textiles. EOs such as citronella, lemongrass, rosemary, peppermint, holy basil, tea tree, neem, lavender, thyme, lemon eucalyptus, clove, and cinnamon have shown insect-repellent activity.

Divan Coetzee [204] reviewed advances in insect-repellent textiles using EO-loaded biopolymer microcapsules. Two main topics were addressed: (1) comparisons of natural vs. synthetic insect repellents, and (2) the roles of key EO components in repellent activity. They found that natural EOs generally performed better in their complete form, rather than as isolated compounds. Immobilized EOs were justified when long-lasting repellency was required, although achieving adequate fixation and controlled release remains a limitation.

EOs with strong antimicrobial properties—cinnamon, thyme, clove, eucalyptus, and lavender—have been widely studied for application in medical textiles, public transportation, and other environments where microbial contamination is common. Microcapsules are designed to provide controlled release, with shells that protect the volatile EO core from degradation. Moisture from sweat often acts as an external triggering effect, causing capsule swelling and enabling diffusion of the active compound.

Bojana [205] produced microcapsules of sage, lavender, and rosemary EOs via in situ polymerization to functionalize shoe insoles with antimicrobial properties. Melamine–formaldehyde was used as the wall material, with styrene–maleic acid anhydride as a modifying agent. The pressure-responsive microcapsules released EOs under mechanical stress during walking. After 50 km of use, insoles retained 60–70% of the encapsulated EO.

Javid et al. [206] prepared EO microcapsules for textile finishing and compared two surfactants: a biosurfactant (rhamnolipid) and CTAB. They found that biosurfactant-produced microcapsules had a narrower size distribution, while antibacterial activity increased with EO and chitosan amounts up to a certain level.

In summary, EO microencapsulation offers broad possibilities across cosmetic textiles, decontamination fabrics, odor-control textiles, self-cleaning materials, and self-healing fibers.

## 9. Concluding Remarks and Further Research

This review examines essential oil (EO) chemistry, microencapsulation methods, and the mechanisms relevant to imparting biofunctional properties to textile substrates. To support this aim, the outline of the fundamental characteristics of EOs, including their chemical composition and antimicrobial potential, has been incorporated. Particular attention is given to commonly studied EOs such as citronella, clove, cinnamon, lavender, eucalyptol, thyme, and rosemary, which are frequently reported for their antibacterial activity. Terpenes and terpenoids constitute major classes of EO constituents and are responsible for many biological effects, including antimicrobial action. Phenylpropanoids also contribute important antioxidant, antimicrobial, and photoprotective properties, supporting applications across food, pharmaceutical, cosmetic, and textile sectors.

Before exploring the various microencapsulation technologies, chemical approaches, and combinations of both used to create complex capsules for immobilizing EOs and applying them to fabric to achieve new functionalities, their manufacturing processes pose significant challenges. These involve two main steps: selecting the wall material for the shell and choosing the method to immobilize the EO. A variety of raw materials are available, including both natural and synthetic options. Although synthetic polymers are most commonly used because of their stability, they are not biodegradable. The production of bio-based microcapsules, such as cyclodextrin inclusion complexes, liposome complexes, gelatin-based, alginate, and cellulose-based microcapsules, can endow textile substrates with biofunctional properties. However, chitosan remains the most frequently used biopolymer as a shell material due to several reasons. It is cheaper, more readily available, and its polarity is vital for fighting environmental microorganisms. Chitosan can be combined with various biopolymers and satisfies all industry requirements, including scalability.

Afterwards, selecting the methodology for immobilized EO is the second step. There is a wide range of processes available to manufacture complexes that immobilize EO and protect its active compounds.

However, the review focuses on biodegradable compounds, in line with the sustainability criteria. When sustainability criteria are considered—particularly the need to avoid synthetic polymers and solvent-intensive processes—only a limited number of methods remain suitable. Coacervation, in both its simple and complex forms, is a promising technique because it enables EO encapsulation using biopolymers and offers scalability compatible with industrial deployment.

Translating laboratory-scale advances into viable products requires alignment with industrial and commercial needs, with a focus on durability, safety, cost, and consumer acceptance.

The limited ability to predict microcapsule morphology and particle size, parameters that significantly influence performance on textile surfaces, is a future challenge. A related limitation is the study of how fabric porosity affects capsule adhesion, release, and durability.

While high-value applications such as healthcare and aviation can support premium technologies, broader adoption in public health or mass-market textiles requires cost-effective encapsulation systems. This underscores the need to explore alternative surfactants, natural polymers, and scalable encapsulation technologies.

The post-pandemic increase in interest in antimicrobial materials further highlights the need for sustainable, affordable, and industrially scalable EO-based systems. Continued research into efficient encapsulation methods will be essential for integrating biofunctional EOs into commercially viable textile products.

## Figures and Tables

**Table 1:** Major organic compounds from chosen EOs with emphasis on antibacterial effect.

Major Org. Compound	Cinnamon	Clove	Lavender	Rosemary	Eucalyptus	Citronella	Chemical Class
Cinnamaldehyde	✓						Phenylpropanoid (aldehyde)
Eugenol	✓	✓					Phenolic phenylpropanoid
Linalool			✓				Monoterpene alcohol
Linalyl acetate			✓				Monoterpene ester
1,8-Cineole (Eucalyptol)				✓	✓		Monoterpene oxide
Camphor				✓			Monoterpene ketone
α-Pinene				✓	✓		Monoterpene hydrocarbon
Citronellal						✓	Monoterpene aldehyde
Geraniol						✓	Monoterpene alcohol
Citronellol						✓	Monoterpene alcohol

**Table 2:** MIC and mechanism of action of some EOs against Gram-positive and negative bacteria.

Essential Oil	Mechanism of Action	MIC vs. Gram+ (mg/mL)	MIC vs. Gram− (mg/mL)	Relative Potency
*Cinnamomum zeylanicum* (Cinnamon)	Disruption of the cell membrane inhibits key metabolic enzymes	0.05–0.5	0.1–1.0	Very High [28]
*Syzygium aromaticum* (Clove)	Membrane permeabilization, leakage of intracellular contents	0.1–0.8	0.2–1.5	Very High [27]
*Thymus vulgaris* (Thyme)	Disrupts lipid bilayer	0.06–0.6	0.1–1.0	Very High [28]
*Rosmarinus officinalis* (Rosemary)	Moderate membrane perturbation, oxidative stress induction	0.5–5.0	1.0–8.0	Moderate [27]
*Cymbopogon nardus* (Citronella)	Membrane destabilization, alteration of fatty acid composition	0.2–2.0	0.5–3.0	High [32]
*Lavandula angustifolia* (Lavender)	Disrupts membrane integrity, increases permeability, and mildly inhibits metabolic enzymes	0.5–5.0	1.0–10.0	Moderate [28]

## Data Availability

Data can be delivered by requirement.
